# Conformationally Constrained Sialyl Analogues as New Potential Binders of h‐CD22

**DOI:** 10.1002/cbic.202200076

**Published:** 2022-03-30

**Authors:** Rosa Ester Forgione, Ferran Fabregat Nieto, Cristina Di Carluccio, Francesco Milanesi, Martina Fruscella, Francesco Papi, Cristina Nativi, Antonio Molinaro, Pasquale Palladino, Simona Scarano, Maria Minunni, Marco Montefiori, Monica Civera, Sara Sattin, Oscar Francesconi, Roberta Marchetti, Alba Silipo

**Affiliations:** ^1^ Department of Chemical Sciences University of Naples Federico II Via Cintia 4 80126 Napoli Italy; ^2^ Department of Chemistry “Ugo Schiff” University of Florence Polo Scientifico e Tecnologico 50019, Sesto Fiorentino Firenze Italy; ^3^ Centro Risonanze Magnetiche CERM Via L. Sacconi 6 50019 Sesto Fiorentino Firenze Italy; ^4^ Dipartimento di Chimica Università degli Studi di Milano via C. Golgi, 19 20133 Milano Italy

**Keywords:** glycans, h-CD22, molecular recognition, NMR spectroscopy, Siglecs

## Abstract

Here, two conformationally constrained sialyl analogues were synthesized and characterized in their interaction with the inhibitory Siglec, human CD22 (h‐CD22). An orthogonal approach, including biophysical assays (SPR and fluorescence), ligand‐based NMR techniques, and molecular modelling, was employed to disentangle the interaction mechanisms at a molecular level. The results showed that the Sialyl‐TnThr antigen analogue represents a promising scaffold for the design of novel h‐CD22 inhibitors. Our findings also suggest that the introduction of a biphenyl moiety at position 9 of the sialic acid hampers canonical accommodation of the ligand in the protein binding pocket, even though the affinity with respect to the natural ligand is increased. Our results address the search for novel modifications of the Neu5Ac‐α(2‐6)‐Gal epitope, outline new insights for the design and synthesis of high‐affinity h‐CD22 ligands, and offer novel prospects for therapeutic intervention to prevent autoimmune diseases and B‐cell malignancies.

## Graphical abstract

**The h‐CD22 ability** to regulate the immune system has made this receptor an attractive therapeutic target. Here we report the characterization of the binding epitopes and affinities of two synthetic analogues bound to h‐CD22 together with the determination of tridimensional models of the protein‐ligand complexes.

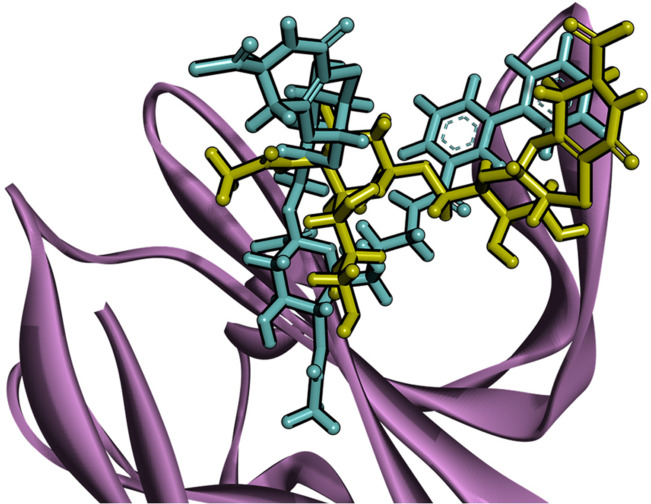

## Introduction

CD22 (Siglec‐2) is a transmembrane receptor belonging to the evolutionary conserved class of Siglecs (Sialic acid binding immunoglobulin type lectins) and is selectively expressed on B‐lymphocytes and, to a lesser extent, to other hematopoietic system cells.[^1^, ^2^, ^3^]

Upon sialoglycans engagement, CD22 negatively modulates B Cell Receptor (BCR) signaling with significant implications in maintaining tolerance to self‐antigens, mandatory to prevent autoimmune diseases and B cells related malignancies.[^4^, ^5^] The mechanism of BCR modulation involves the formation of CD22 homo‐oligomers on resting B cells by means of *cis* interactions. Subsequently, specific antigens provoke a conformational change of BCR, leading to the activation of immune response and to the recruitment of CD22 clusters; ultimately, *trans* sialoglycan binding of autologous *self*‐cells triggers BCR inhibition.^[6]^

The accommodation in the binding pocket of CD22 of sialic acids α‐(2‐6)‐linked to galactose epitopes (Neu5Acα(2‐6)‐Gal), common terminus of mammalian surface glycoproteins, has been recently described and occurs with a K_D_=281±10 μM.^[7]^ Furthermore, the conformational behavior of natural complex‐type biantennary *N*‐glycans interacting with CD22 was undertaken^[8]^ thus proving that the terminal Neu5Acα(2‐6)‐Gal is the sole epitope recognized by and interacting with CD22, differently from other lectins‐glycans interactions,^[9]^ and also showing the key role of glycan conformation in triggering CD22 homo‐oligomers formation.

Given its role in health and diseases, therapeutic tools based on CD22 as candidate target in immunomodulation and tumor therapies have been conceived, particularly focusing on the development of antibody‐based approaches.[^10^, ^11^, ^12^] Nonetheless, alternative lines are also under evaluation, including glycan‐based therapies that rely on the design of high affinity ligands with the capability to take over endogenous glycans.

In this context, low‐molecular‐weight Siglec ligands and their conjugates, especially those based on synthetic sialic acid derivatives, showed promising results in clinical trials as therapeutics for the nerve regeneration, oncology, and immunology.^[13]^ The majority of sialic acid analogues are characterized by the modifications of functional groups, as at carbons 2, 4, 5, and 9. In particular, substitutions at the hydroxyl/*N*‐acetyl groups at position 9 and 5 respectively have shown to significantly enhance the binding affinity to Siglecs.[^13^, ^14^, ^15^, ^16^] In parallel, the design of polyvalent constructs that include Siglecs specific‐ high affinity sialylated probes, to be conjugated to multivalent carriers, is a further step for biomedical applications.[^13^, ^14^, ^15^, ^16^, ^17^]

In a recent paper,^[18]^ the synthesis and biological activity of a Neu5Ac‐α(2‐6)‐Gal derivative, developed as an analogue of the Sialyl TnThr tumor antigen, were reported. From the structural viewpoint, the synthetic compound is a constrained tricyclic glycoside, that retains the α‐*O*‐glycosidic linkage at the Gal moiety and in which the terminal lactam ring mimics the Thr residue of the Tn/STn natural antigens. The Sialyl TnThr analogue belongs to a class of compounds exhibiting interesting inhibitory activity also toward other biological targets.[^19^, ^20^]

With the believe that the rigid galactoside moiety would affect the binding specificity of CD22, here we report the description of the molecular recognition by human CD22 of the Sialyl‐TnThr analogue and of a newly synthesized derivative, with the aim to identify novel ligands with therapeutic and diagnostic potential. The binding mode of such sialylated analogues was depicted by using NMR, fluorescence, Surface Plasmon Resonance (SPR), and molecular modelling techniques. The obtained results might contribute to rational design of CD22 potent inhibitors and/or regulators.

## Results and Discussion

**Figure 1:**
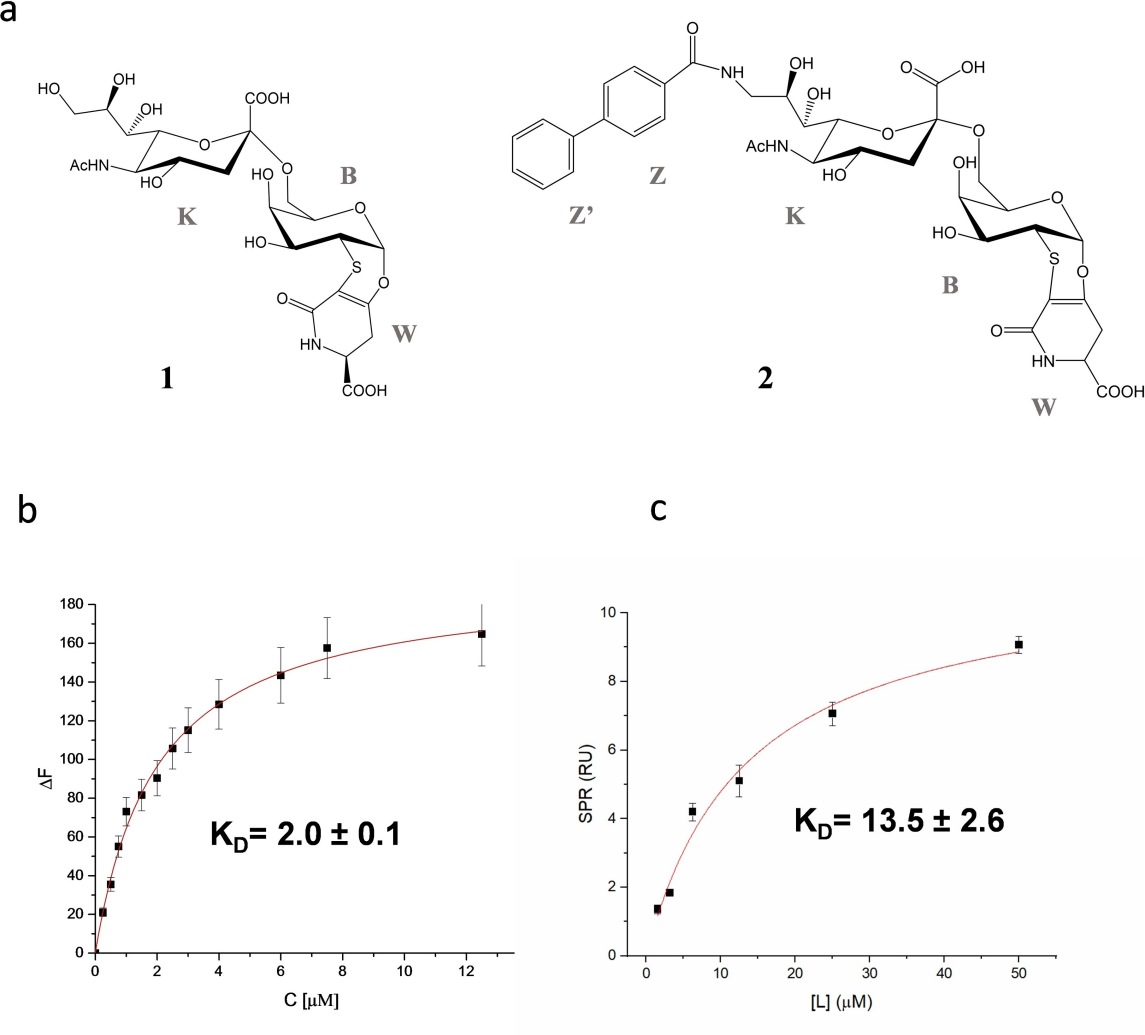
Binding affinity of h‐CD22 and sialic acid analogues. a) Structures of the sialic acid analogues used in this study. b) Fluorescence titration of h‐CD22 upon the addition of analogue **1**. Each emission spectrum was recorded at the excitation wavelength of 285 nm and a temperature of 10 °C. The relative binding isotherm and the value of the dissociation constant (K_D_) is reported. For each data point, 10 % Y error bars are shown. c) SPR binding curve for analogue **2** vs h‐CD22 on Protein A. Each point is representative of 3 replicates (RU_mean_±SD).

Based on the structure of the previously synthesized sialo‐derivative **1** (Figure 1a),[^18^, ^19^, ^20^] the synthesis of **2** has been conceived. The sialo‐derivative **2** (Figure 1a), having a similar scaffold than **1** but bearing a biphenyl substituent at position 9, was synthetized based on the observation that a similar sialo‐analogue increased the bound potency and selectivity for CD22.^[21]^

**Scheme 1:**
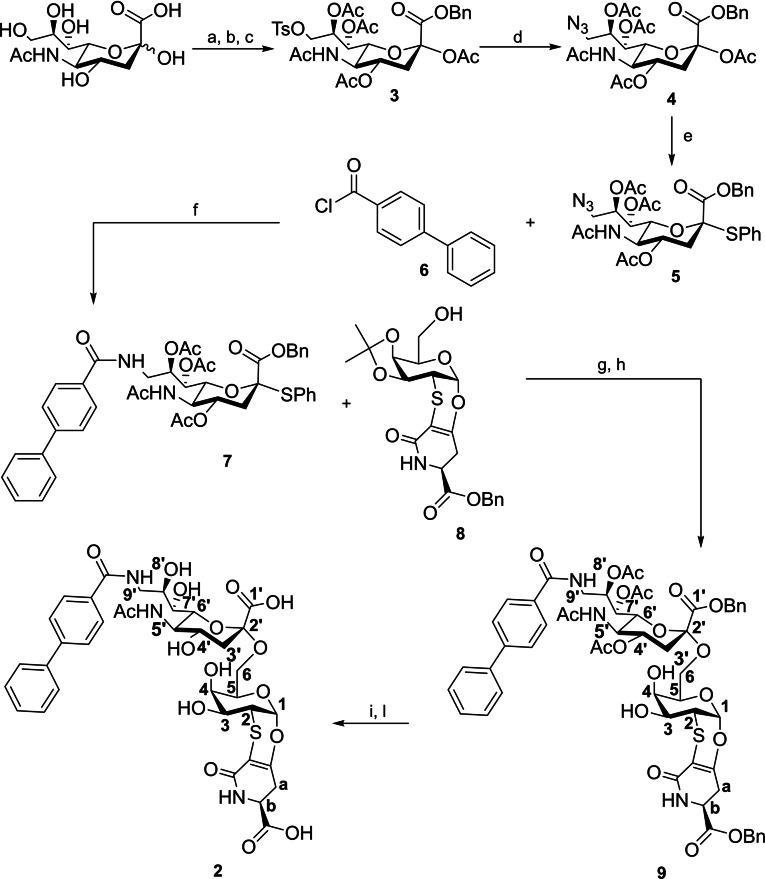
Synthesis of sialo derivative **2** with atom labelling. Reagents and conditions: (a) BnBr, DBU, DMF, room temperature, 18 h; (b) TsCl, Py, room temperature, 18 h; (c) Ac_2_O, DMAP, Py, room temperature, 5 h; (d) NaN_3_, dry DMF, 70 °C, 5 h; (e) PhSH, BF_3_⋅Et_2_O, dry DCM, room temperature, 18 h; (f) PPh_3_, CH_2_Cl_2_, room temperature, 48 h; (g) NIS, TfOH, dry CH_3_CN/CH_2_Cl_2_ (10 : 1), −40 °C, 4 h; (h) AcOH 80 %, 40 °C, 18 h; (i) Pd/C, H_2_, MeOH, room temperature, 72 h; (l) NH_3_ 4 M in MeOH, room temperature, 120 h.

In detail, analogue **2** was prepared (see Scheme 1) starting from *N*‐acetylneuraminic acid. Esterification of the carboxylic group using benzyl bromide was followed by tosylation of the hydroxylic group in position 9 and acetylation of the remaining positions to give the protected sialic derivative **3** with a 40 % yield on three steps. Tosylate group of **3** was then substituted with NaN_3_ to give the azido derivative **4** with a 50 % yield. Reaction of **4** with thiophenol in presence of BF_3_⋅Et_2_O gives the *S*‐phenyl sialoside **5** with a 92 % yield. Intermolecular Staudinger ligation between **5** and [1,1′‐biphenyl]‐4‐carbonyl chloride **6** ^[22]^ in presence of PPh_3_ gave the biphenyl substituted sialoside **7** with a 30 % yield. Glycosylation reaction between the glycosidic donor **7** and the acceptor **8**, prepared according to known literature,^[23]^ was carried out activating the *S*‐phenyl group of **7** with *N*‐iodosuccinimide (NIS) and catalyzing the reaction with **8** using TfOH. After removal of the isopropylidene group with AcOH 80 %, the sialo derivative **9** was obtained with a 40 % yield in two steps. Removal of the benzyl ester by hydrogenation and of the acetylic groups with an ammonia solution in methanol, gave the sialo derivative **2** with a yield of 30 % on two steps.

### Molecular recognition of a sialic acid analogue 1 by CD22

The binding features of the sialo‐derivative **1** (Figure 1a) in the interplay with h‐CD22 were described by means of fluorescence studies, ligand‐based NMR techniques and molecular modelling.

Intrinsic fluorescence studies of analogue **1** in the interaction with h‐CD22 showed that the tryptophane residues of the receptor were quenched by the ligand addition, thus proving the complex formation. The interpolation of the fluorescence data provided the corresponding dissociation constant K_D_=2.0±0.1 μM (Figure 1b and Figure S13).

**Figure 2:**
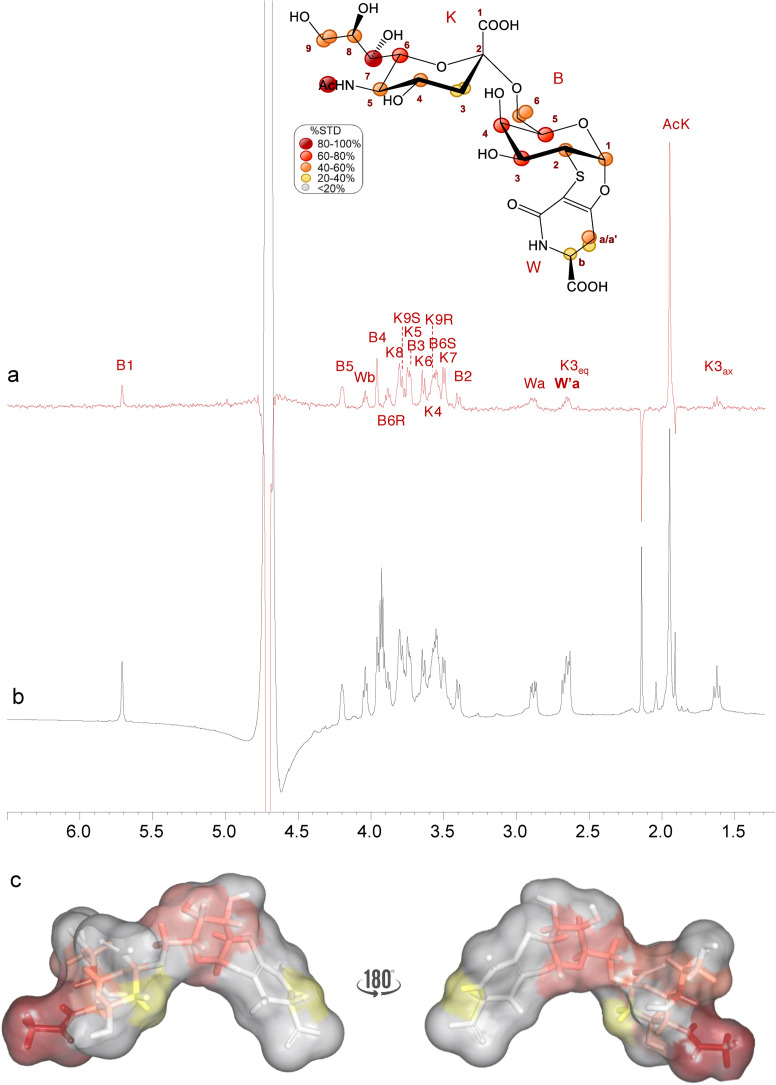
STD NMR analysis of analogue **1** in the interaction with h‐CD22. Superimposition of the STD NMR spectrum (a) and the ^1^H NMR spectrum (b) of h‐CD22/analogue **1** mixture with a molecular ratio of 1 : 100, at 298 K. The interacting epitope map of analogue **1** as derived by STD‐NMR data is also reported. c) 3D representation of the analogue **1** in the bioactive conformation obtained by tr‐NOESY with molecular surface colored according to STD enhancements.

Furthermore, STD NMR[^24^, ^25^] and tr‐NOESY^[26]^ have been employed to dissect at molecular level the interaction between analogue **1** and h‐CD22 and to map the ligand's interacting epitope and bioactive conformation (Figure 2). The STD NMR spectrum highlighted that the sialyl derivative was recognized by h‐CD22 (Figure 2) as shown by the relative enhancements in the STD spectrum: a strong contribution from the neuraminic acid (Neu5Ac) moiety was detected, with the highest STD effect observed for its acetyl group. Furthermore, the involvement of the Neu5Ac lateral chain was deduced from the strong enhancements (between 50 % and 70 %) observed for K5, K6, K7, K8 and K9_R/S_ protons. Significant STD effects were observed also for the galactose (Gal) residue, in particular for protons B3, B4 and B5. Interestingly, also the diastereoisotopic protons of the lactam moiety Wa/a’ showed low STD signals, thus suggesting that also the aglycon moiety was involved in the binding.

With the aim to explore the dynamic behavior of the ligand into the h‐CD22 binding site, NOE‐based experiments were combined with molecular dynamics (MD) studies (Figure S14). The analogue **1** exhibited a similar conformational behavior in free and bound states, as evident by comparing the NOE and tr‐NOE contacts. In detail, ϕ torsion angle around the glycosidic linkage between Neu5Ac‐α‐(2‐6)‐Gal was located around −60° (Figure S14a,S15b), as demonstrated by the absence of the NOE contacts between B6 and K3 (Figure S14c/d) and confirmed by the stability of MD trajectories in both free and bound states (Figure S15). Despite a similar conformational behavior, analogue **1** adopted a more extended conformation due to the rigid nature of the lactam ring (W) if compared to 6’SLN umbrella‐like topology.^[27]^

**Figure 3:**
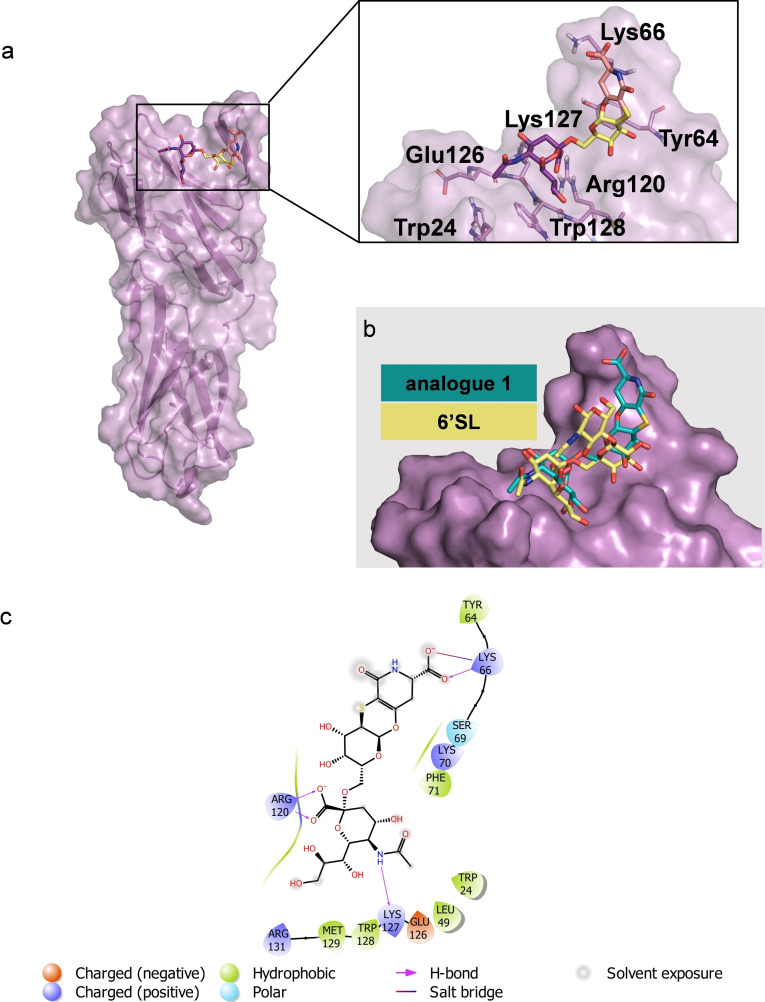
Interaction between h‐CD22 and analogue **1** by molecular modelling. a) 3D model derived by docking and MD simulations for the analogue **1** bound to h‐CD22 (PDB ID: 5VKM). The representative frame of the most populated MD cluster, obtained by Kmeans algorithm, was considered to depict the complex. b) Superimposition of the previously obtained X‐ray complex of h‐CD22/6’sialyllactose (6’SL) and the analogue **1** bound model. c) Two‐dimensional plots representing the interactions between the analogue **1** and the binding site residues of h‐CD22.

Computational studies also allowed to construct a 3D model of the protein‐ligand complex (Figures 3, S16). Docking calculations showed that in the most populated binding mode, the analogue **1** is accommodated in the canonical binding site of h‐CD22 (Figure 3). Such docked pose was then considered as starting geometry for MD simulations. The root‐mean square deviation (RMSD) analysis showed that the ligand remained stable in the binding pocket of the receptor for the entire simulation time (Figure S15a). The analysis of the interactions of representative models obtained from MD trajectory clustering highlighted that the sialic acid moiety interacted with Arg120 to form the carboxylate bridge essential determinant for Siglecs’ recognition,^[4]^ and that this interaction was maintained for 100 % of the simulated time. Moreover, the lateral glycerol protons of Neu5Ac chain established stacking interactions with Trp128 residue, as well as hydrogen bonds with Arg131. Additionally, the *N*‐acetyl group formed a hydrogen bond with Lys127. The Gal pyranose ring stacked with the Tyr64 residue thus explaining the significant STD effects. Notably, the aglycon moiety was in contact with the CC’ loop of h‐CD22 and engaged a strong hydrogen bond between its carboxylate group and the Lys66 residue of the loop, implying a significant involvement of W moiety in the binding without remarkable alteration of the minimally recognized epitope.

### Molecular recognition of sialic acid analogue 2 by CD22

The binding features of the sialo‐derivative **2** (Figure 1a) were investigated in the interaction with h‐CD22 by means of SPR, NMR and molecular modelling.

In detail, the interaction between a dilution series of **2** in HBS‐EP buffer and the CD22 protein immobilized on a working cell of SPR gold chip modified with protein A was evaluated against a reference cell with only protein A to compensate for matrix interferences, refractive index effects and non‐specific binding of the analyte (see Experimental Section). Small but reproducible SPR signals were fitted according to a 1 : 1 binding model (Figure 1b), indicating an apparent dissociation constant in the micromolar range (K_D_=13.5±2.6 μM). Differently, no significative SPR signals were detected upon application of the same method to sialo‐derivative **1**. We tentatively ascribe these different results to the limitations of the applied method that resulted sensitive enough for to sialo‐derivative **2** only, probably for the higher hydrophobicity and larger mass of **2** with respect to sialo‐derivative **1** (802.21 *vs* 623.14 g mol^−1^).

**Figure 4:**
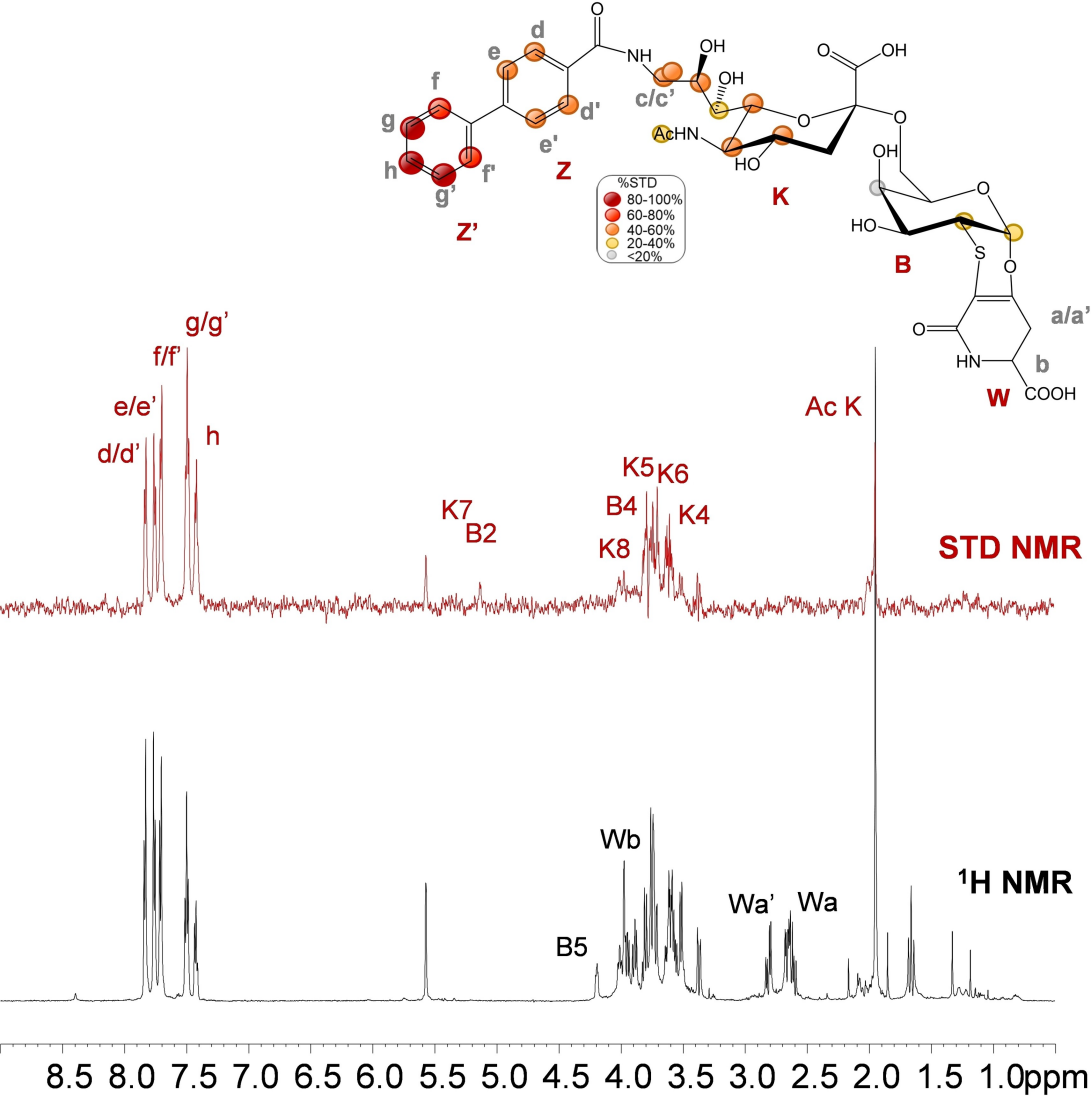
STD NMR analysis of analogue **2** in the interaction with CD22. Interacting epitope map of analogue **2** as derived by STD‐NMR data (top panel). ^1^H NMR and STD NMR spectra of h‐CD22/analogue **2** mixture with a molecular ratio of 1 : 100, at 298 K (bottom panel).

The interacting epitope of **2** upon h‐CD22 binding was described by STD NMR (Figure 4). Interestingly remarkable STD enhancements from the aromatic moiety were detected, suggesting a major involvement of the biphenyl ring in the recognition process. The highest STD effect belonged to the Z’ aromatic ring of the biphenyl group with the intensity of proton **h** at 100 %, although considerable STD enhancements were detected also for the Z ring (between 40–60 %). Differently from **1**, the aglycon moiety on the Gal unit did not contribute to the binding event. Concerning the saccharide moieties, very weak STD effects were observed for H1, H2, H4 protons of galactose unit (**B**). Moreover, medium (40‐60 %) STD effects were detected for Neu5Ac (**K**) unit, specifically for H4, H5, H6, H8, H9_R/S_ protons. Low STD effects were observed for H7 and the *N*‐acetyl group of Neu5Ac residue. This suggested that, conversely from the natural ligand, the recognition is not driven by the Neu5Ac unit, but rather by the biphenyl moiety.

**Figure 5:**
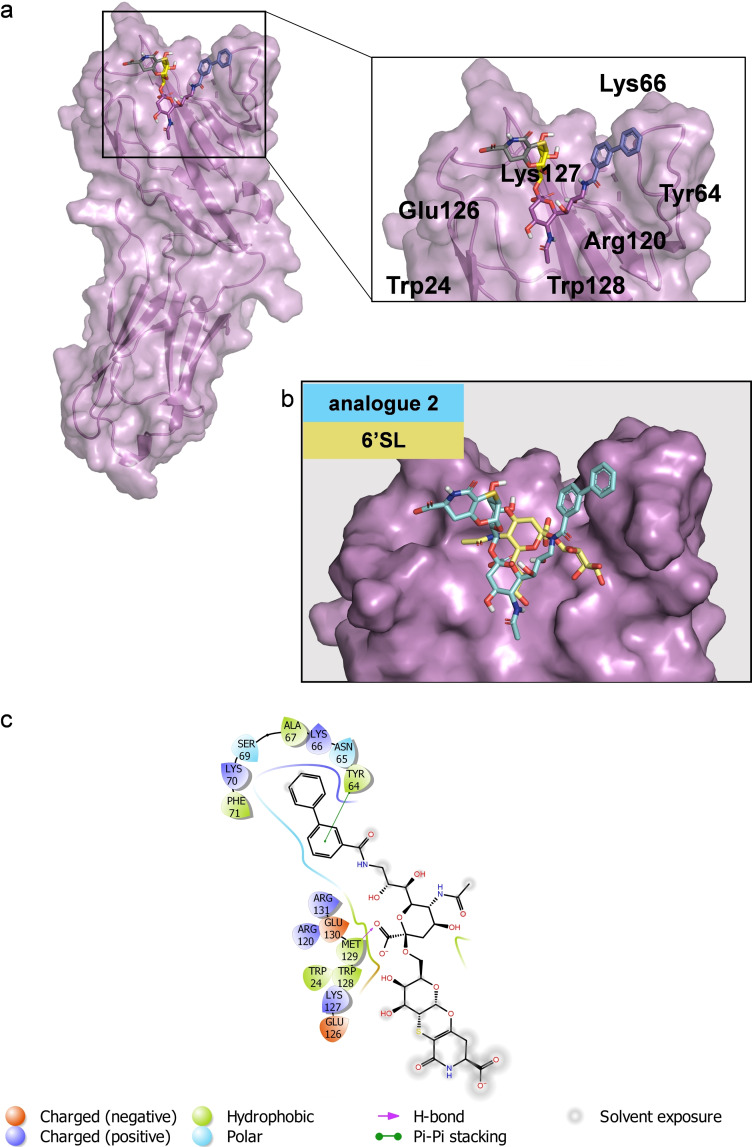
Interaction between CD22 and analogue **2** by molecular modelling. a) 3D model derived by docking calculations of **2** bound to h‐CD22 (PDB ID: 5VKM). The lowest energy cluster binding mode was considered to depict the complex. b) Superimposition of the previously obtained X‐ray complex of h‐CD22/6’SL and the analogue **2** bound model. c) Two‐dimensional plots representing the interactions between the analogue **2** and the binding site residues of h‐CD22.

**Figure 6:**
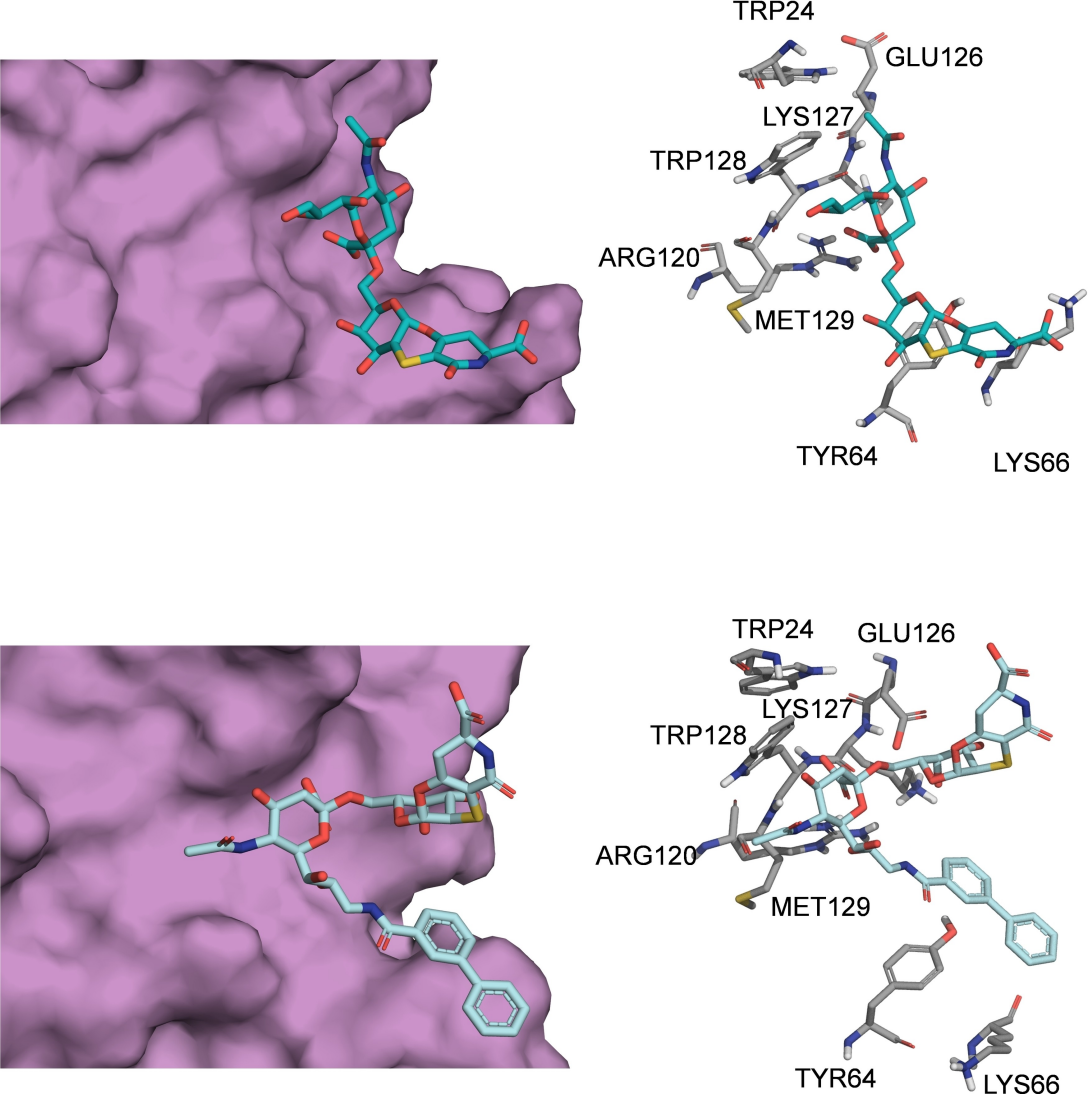
Comparison of the binding modes of analogues **1** and **2** upon interaction with h‐CD22. Close up view of the analogues **1** (light blue sticks) and **2** (cyan sticks) into the h‐CD22 binding site (violet molecular surface). The h‐CD22 residues binding to the ligands are represented as grey sticks.

These data were in agreement with the CD22/ **2** complex (Figure 5) obtained by docking calculations. Interestingly, a close‐up view of the less energetic cluster (Figures 5 and S16b) highlighted that **2** was placed at the protein V‐set domain, directing the biphenyl group toward the CC’ loop of the receptor, stacked against Lys66 and Tyr64 residues of the loop. The Neu5Ac displayed few polar contacts with Arg120, Trp24 and Trp128 whereas the *N*‐acetyl moiety was not oriented toward the canonical hydrophobic cleft comprising Trp24 and Trp128 and lacked the hydrogen bond with Glu126. Interestingly, the orientation of **2** was different from that observed for the natural sialoglycans and for **1** as well (Figure 6). Consistently with the STD‐NMR, Gal and the aglycone moieties were not in contact with the receptor surface. These findings suggested that the presence of the biphenyl moiety, that promoted the favorable stacking interactions at the CC’ loop, leads to a different orientation of the Neu5Ac from its canonical binding site.

## Conclusions

The discovery of the essential roles played by h‐CD22 in the regulation of immune cells and its expression pattern restricted to B cells has made this receptor an attractive therapeutic target.[^28^, ^29^] With the aim to exploit h‐CD22 ability to inhibit specific cells, several strategies have been developed and much efforts have been directed toward the development of CD22 high affinity ligands.[^30^, ^31^, ^32^, ^33^] With the aim to further improve the design of CD22 modulators for therapeutic applications, we studied here two structurally constrained analogues, bearing functionalizable groups at the aglycon moiety allowing for the easy conjugation to bioavailable nanomaterials, in the interaction with h‐CD22.

The characterization of the binding epitopes and affinities together with the determination of tridimensional models of the protein‐ligand complexes have been carried out. In detail, the STD NMR investigation allowed to define the binding epitope of the sialyl derivatives; the bound conformation of the **1** was also described by using NOE based data in combination with a computational approach.

Although the orientation around the Neu5Ac‐α‐(2‐6)‐Gal was the same as observed for natural CD22 ligands[^7^, ^8^, ^34^] also when interacting with other Siglecs, as Siglec‐10,^[35]^ our results suggested a more extended conformation of the analogue **1** if compared to the natural ligand due to the presence of the rigid aglycon moiety. Moreover, docking and modelling studies provided insights into the fine structural characteristics of the interacting interfaces. CD22 bound analogue **1** in the canonical sialic acid binding site, without relevant alterations of the interaction pattern and demonstrated that the rigid aglycone moiety, that was close to the CC’ loop region of CD22 V‐set domain, did not affect the presentation of the Neu5Ac‐Gal epitope to the enzyme.^[7]^

Conversely, the STD‐NMR analysis carried out on **2**, bearing an aromatic extension at C9 of Neu5Ac, indicated that the strongest contribution to the binding was given by this moiety rather than the sialic acid unit itself. Docking studies allowed to identify a good model explaining and supporting the experimental results. Indeed, in the proposed model, the biphenyl group was accommodated at the V‐set domain and oriented toward the CC’ loop region where it formed aromatic interactions with Tyr64. This may be due to the steric hindrance given by the combination of the biphenyl and the aglycon moiety, that makes the sialic acid unit not optimally oriented within the receptor binding pocket, although it still established weaker polar interactions at the CD22 interface.

The interaction between h‐CD22 and the sialo‐analogues **1** and **2** were also proved by SPR and fluorescence assays. Interestingly, the slightly different K_D_ values align with the observations that in **1** the sialyl moiety was well oriented in the receptor binding pocket, without alteration of key interactions established by the natural epitope and with an increased affinity thanks to the contribution of the rigid aglycon moiety. On the other hand, the binding mode of **2** was influenced by the presence of the biphenyl moiety that lead to a different accommodation of the sialic acid in the binding site of the protein with respect to the natural epitope. Despite this, a good increase of binding affinity was observed for **2** with respect to the natural ligand, mostly due to the pi‐pi interactions between the ligand aromatic moiety and the receptor aromatic residues belonging to the CC’ loop. Such interactions were added to the stabilizing interactions of the Neu5Ac moiety with polar the CD22 binding site polar residues.

The obtained results further highlighted the importance of the Siglecs CC’ loop region for the recognition of sialoglycans,^[36]^ thus suggesting that this region may be further exploited to design glycosyl analogues for CD22 immunomodulation and to fine tune its specificity and affinity. Overall, our structural and affinity data showed that the sialyl derivative **1** can strongly interact with h‐CD22; thus, a class of related compounds may be designed as promising candidate for glycoimmunotherapy targeting CD22. Furthermore, the functionalization and characterization of suitable nanomaterials coated with **1**‐like ligands may lead to interesting therapeutic applications, for example to efficiently probe the cell surfaces.[^13^, ^14^, ^15^, ^16^, ^17^, ^18^, ^19^, ^20^, ^21^, ^22^, ^23^, ^24^, ^25^, ^26^, ^27^, ^28^, ^29^, ^30^, ^31^, ^32^, ^33^]

## Experimental Section

Details on protein expression and purification, synthesis, and characterization of chemical materials, SPR, fluorescence, NMR spectroscopy and computational methods can be found in the Supporting Information.

## Conflict of interest

The authors declare no conflict of interest.

1

## Supporting information

As a service to our authors and readers, this journal provides supporting information supplied by the authors. Such materials are peer reviewed and may be re‐organized for online delivery, but are not copy‐edited or typeset. Technical support issues arising from supporting information (other than missing files) should be addressed to the authors.

Supporting Information

## Data Availability

Data sharing is not applicable to this article as no new data were created or analyzed in this study.
