# Supplementary material for: Conformationally Constrained Sialyl Analogues as New Potential Binders of h‐CD22
Source: Chembiochem. 2022 Mar 30;23(10):e202200076. doi: 10.1002/cbic.202200076 (PMC9315041; doi:10.1002/cbic.202200076)
Supplement: Supplementary file 1 — Supporting Information [file CBIC-23-0-s001.pdf]

ChemBioChem

Supporting Information

Conformationally Constrained Sialyl Analogues as New Potential Binders of h-CD22

Rosa Ester Forgione+, Ferran Fabregat Nieto+, Cristina Di Carluccio+, Francesco Milanesi, Martina Fruscella, Francesco Papi, Cristina Nativi, Antonio Molinaro, Pasquale Palladino, Simona Scarano, Maria Minunni, Marco Montefiori, Monica Civera, Sara Sattin, Oscar Francesconi, Roberta Marchetti,* and Alba Silipo

Figures S1-S12. NMR spectra.

Figure S13 Fluorescence spectra of h-CD22 (black line) in the presence of increasing amounts of **1** (colored lines).

Figure S14 Conformational analysis of **1**

Figure S15 MD simulation analysis h-CD22/**1** complex

Figure S16 3D view of the h-CD22/**1** and h-CD22/**2** complexes.

Experimental Procedures

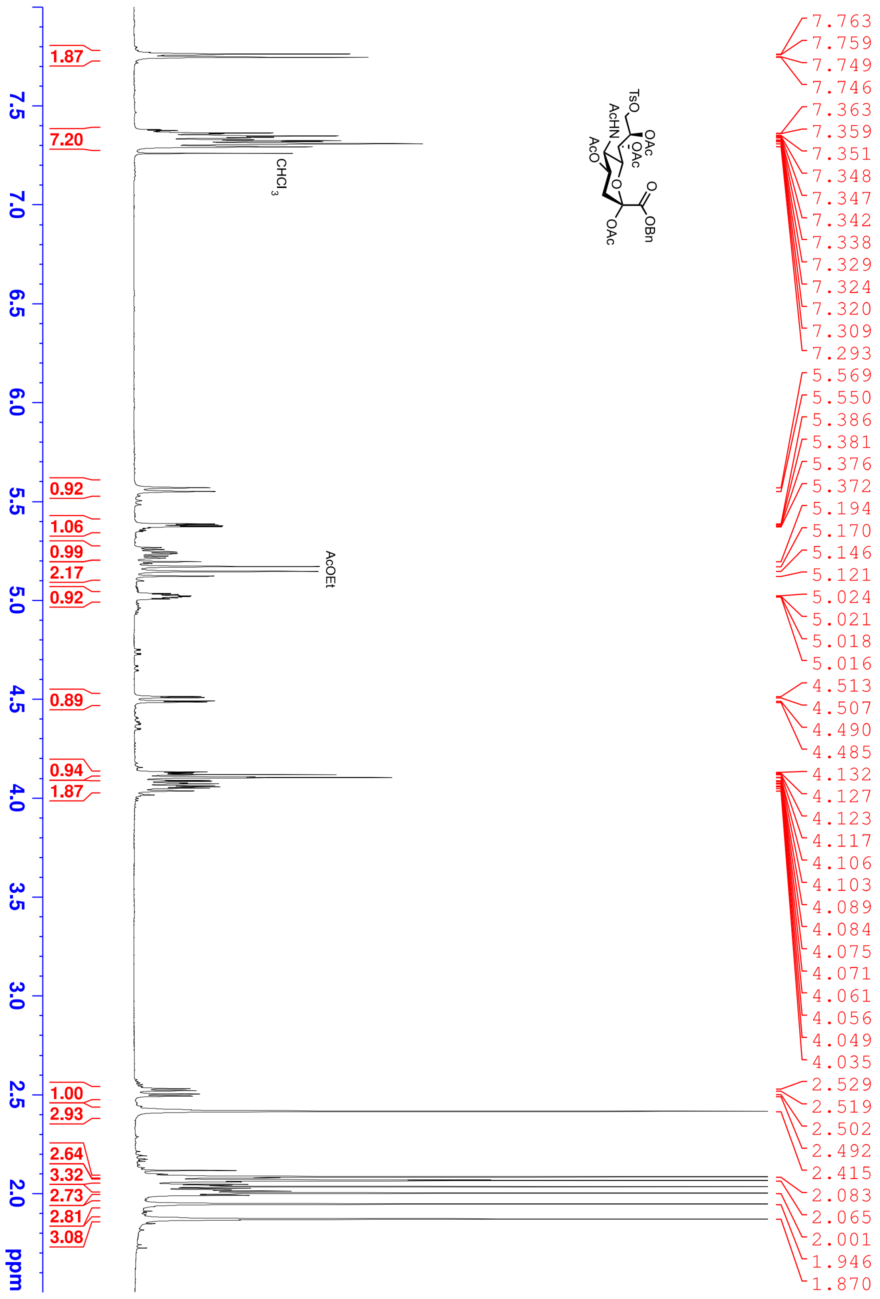

Figure S1. ^{1}H NMR spectrum of **3** (500 MHz, $CDCl_3$).

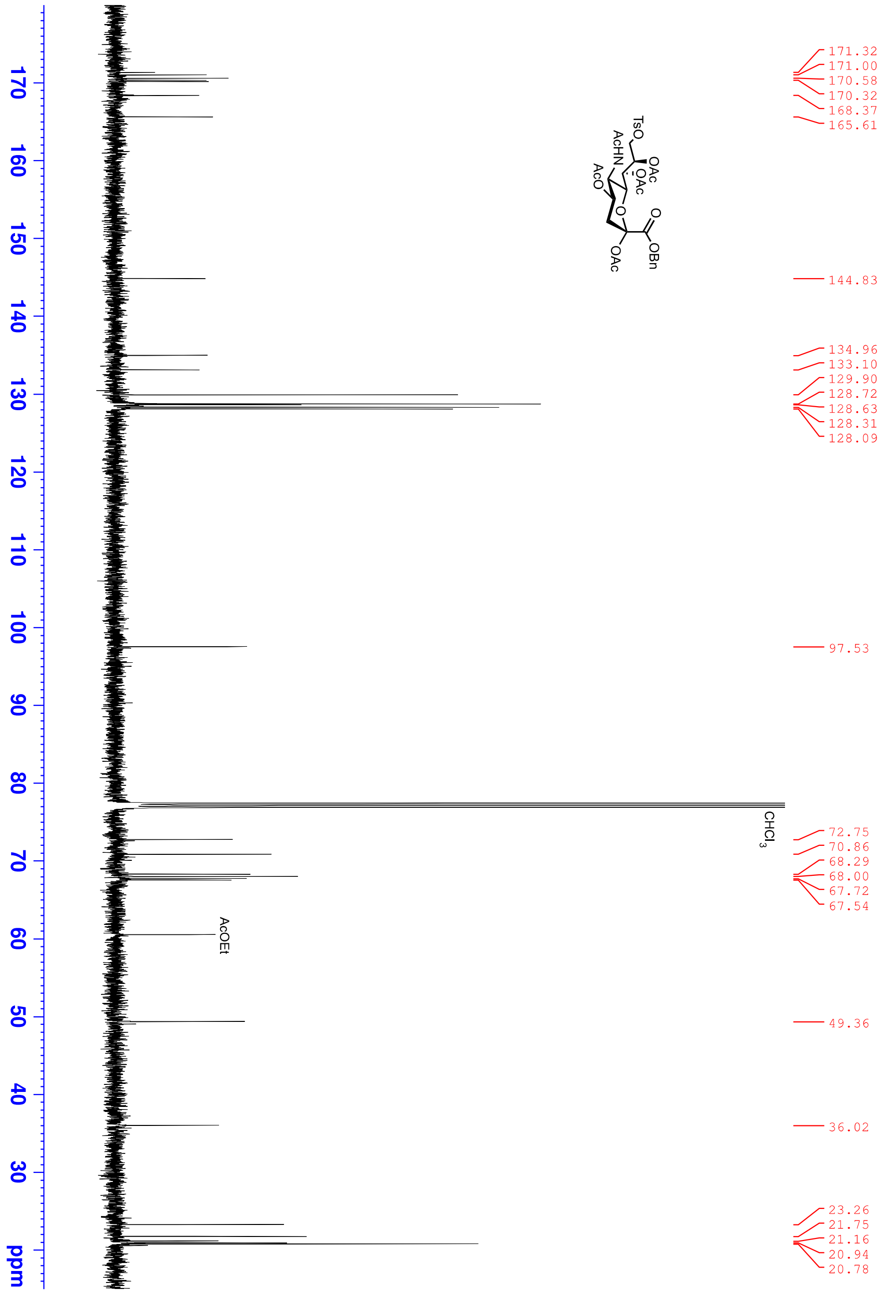

Figure S2. ^{13}C NMR spectrum of **3** (125 MHz, $CDCl_3$).

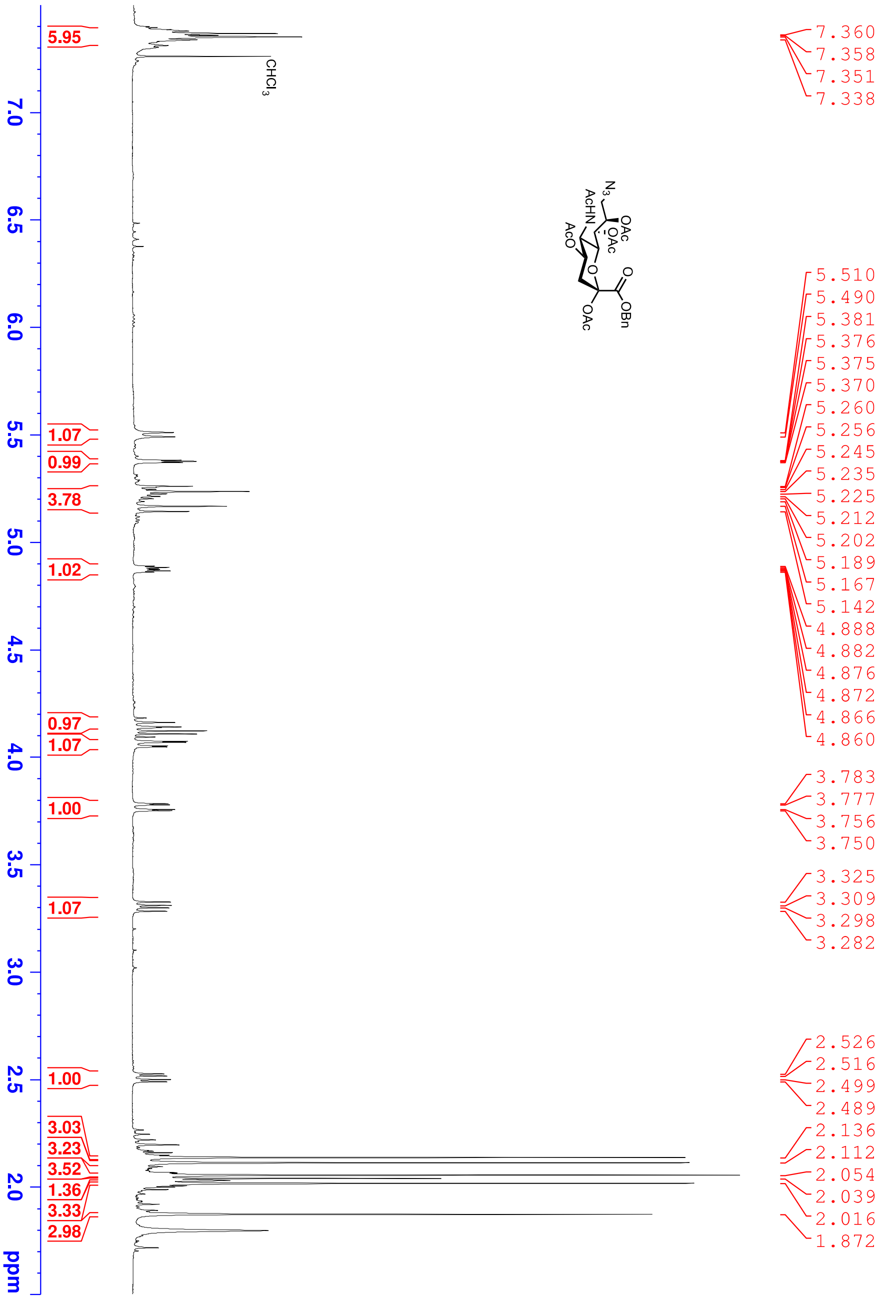

Figure S3. ^{1}H NMR spectrum of **4** (500 MHz, $CDCl_3$).

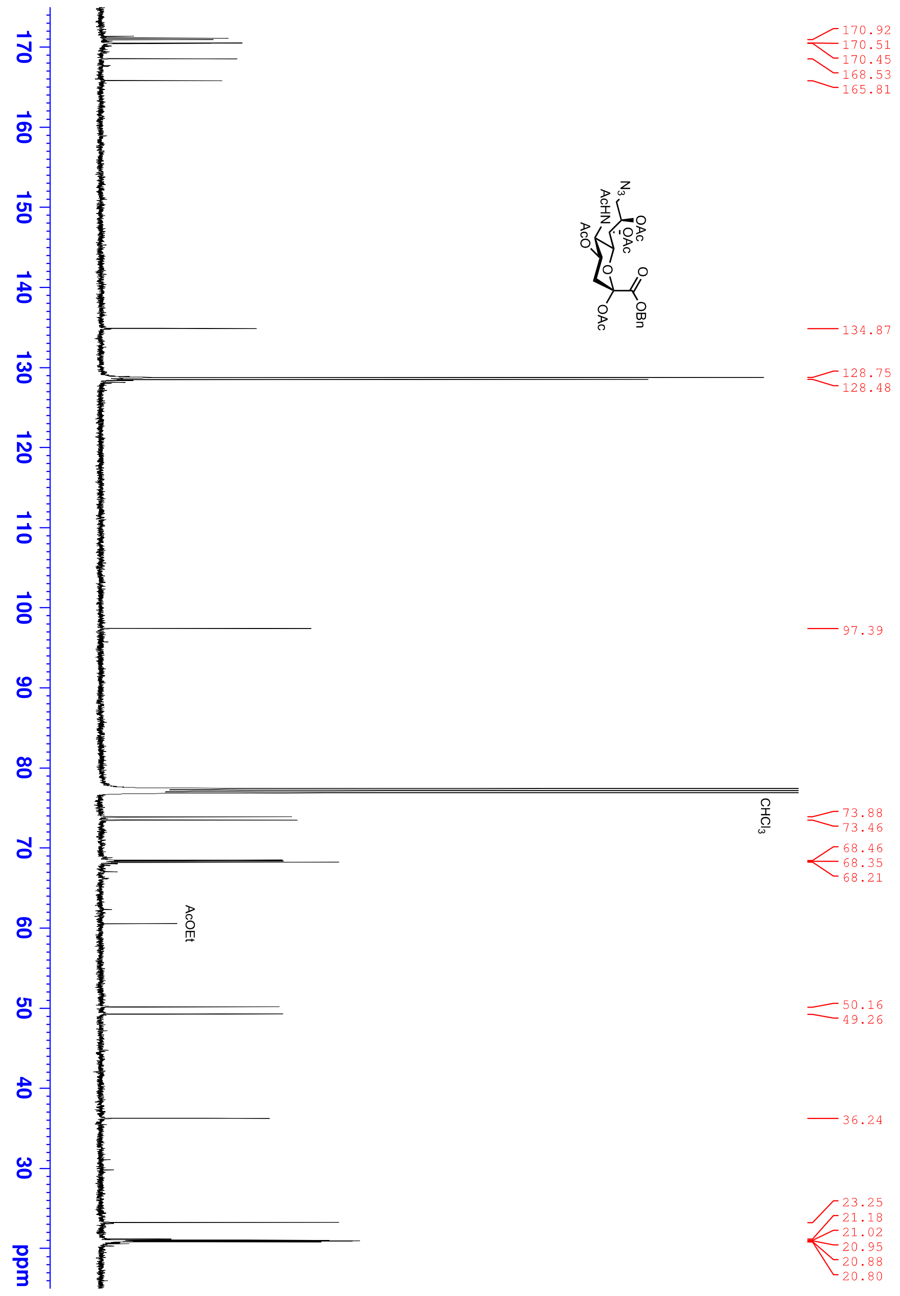

Figure S4. ^{13}C NMR spectrum of **4** (125 MHz, $CDCl_3$).

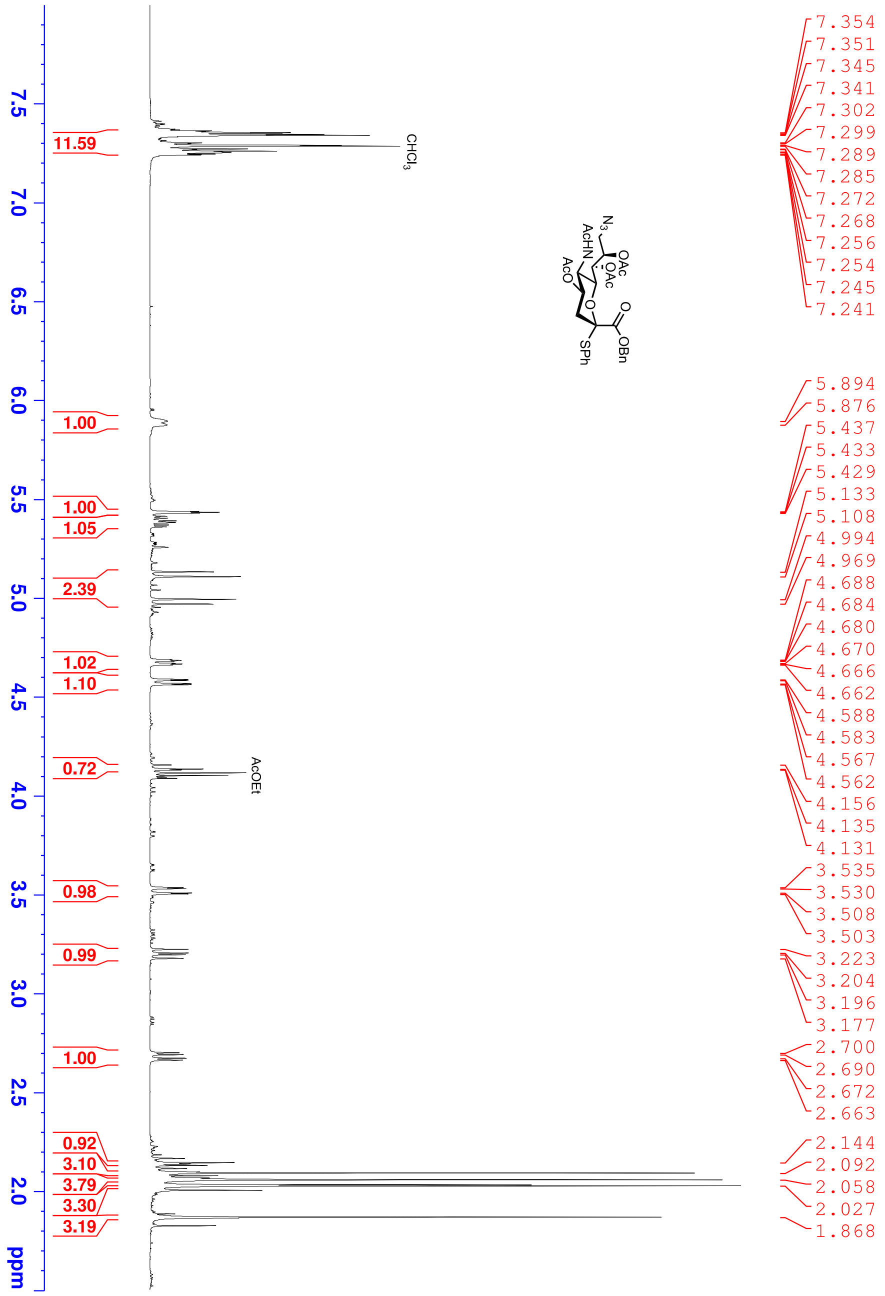

Figure S5. ^{1}H NMR spectrum of **5** (500 MHz, $CDCl_3$).

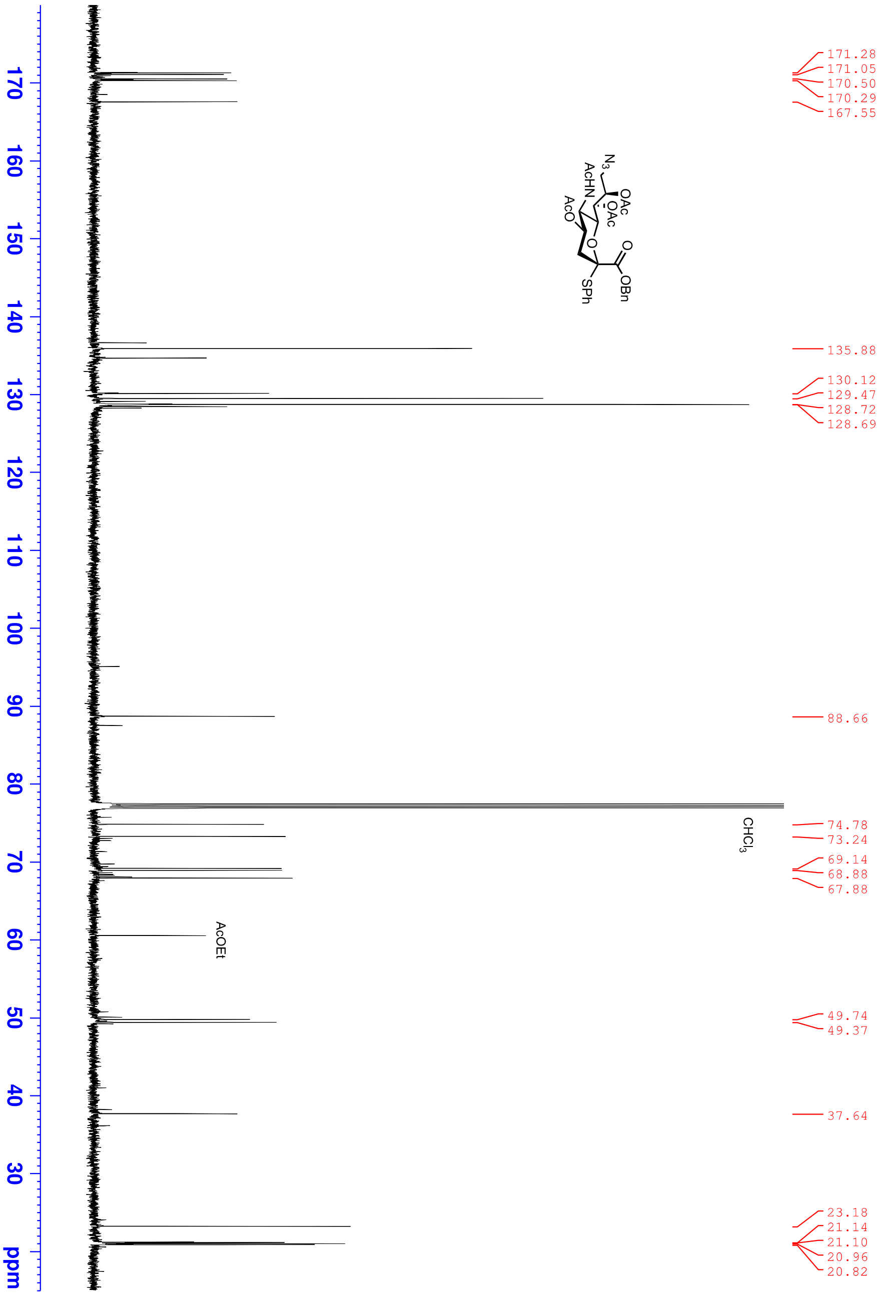

Figure S6. ^{13}C NMR spectrum of **5** (125 MHz, $CDCl_3$).

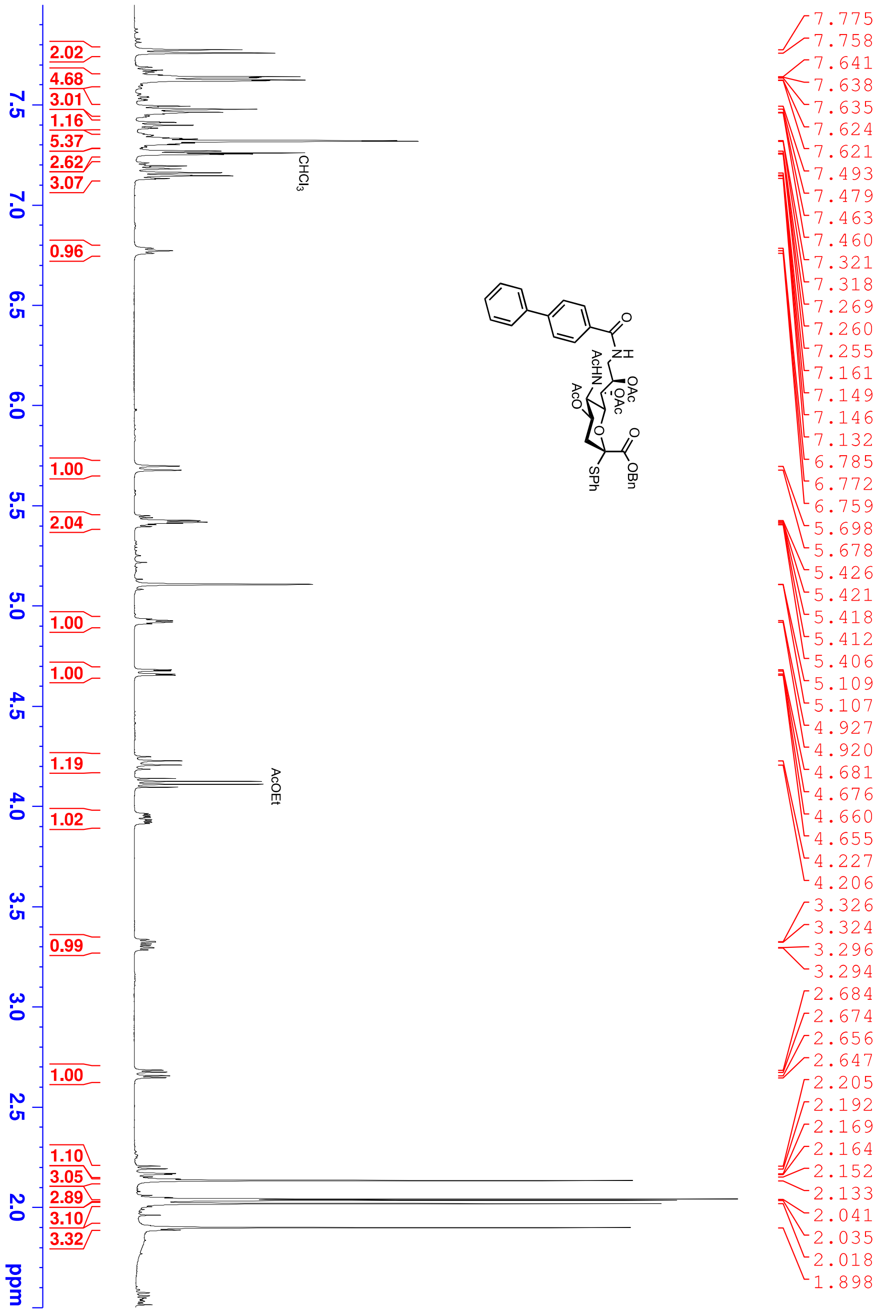

Figure S7. ^{1}H NMR spectrum of **7** (500 MHz, $CDCl_3$).

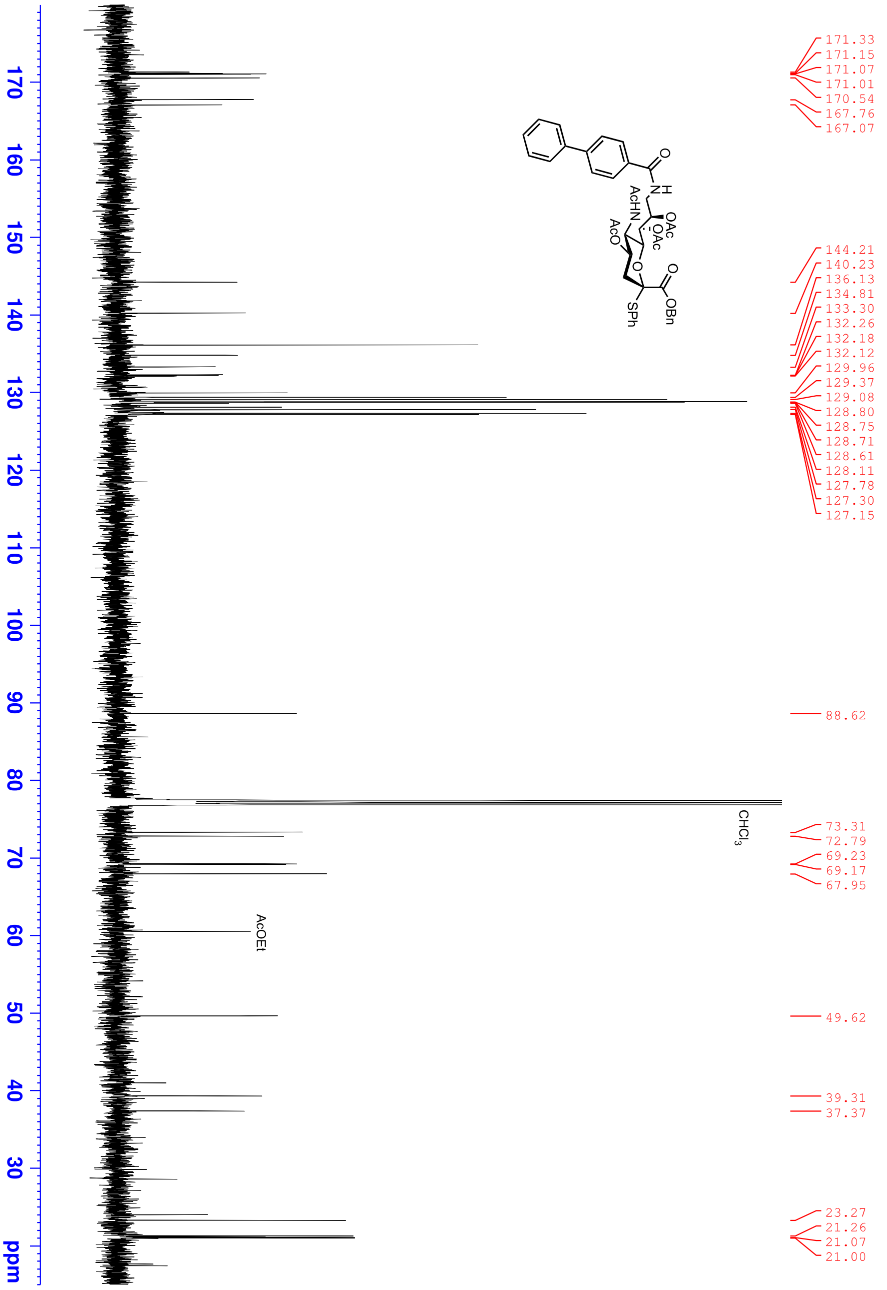

Figure S8. ^{13}C NMR spectrum of **7** (125 MHz, $CDCl_3$).

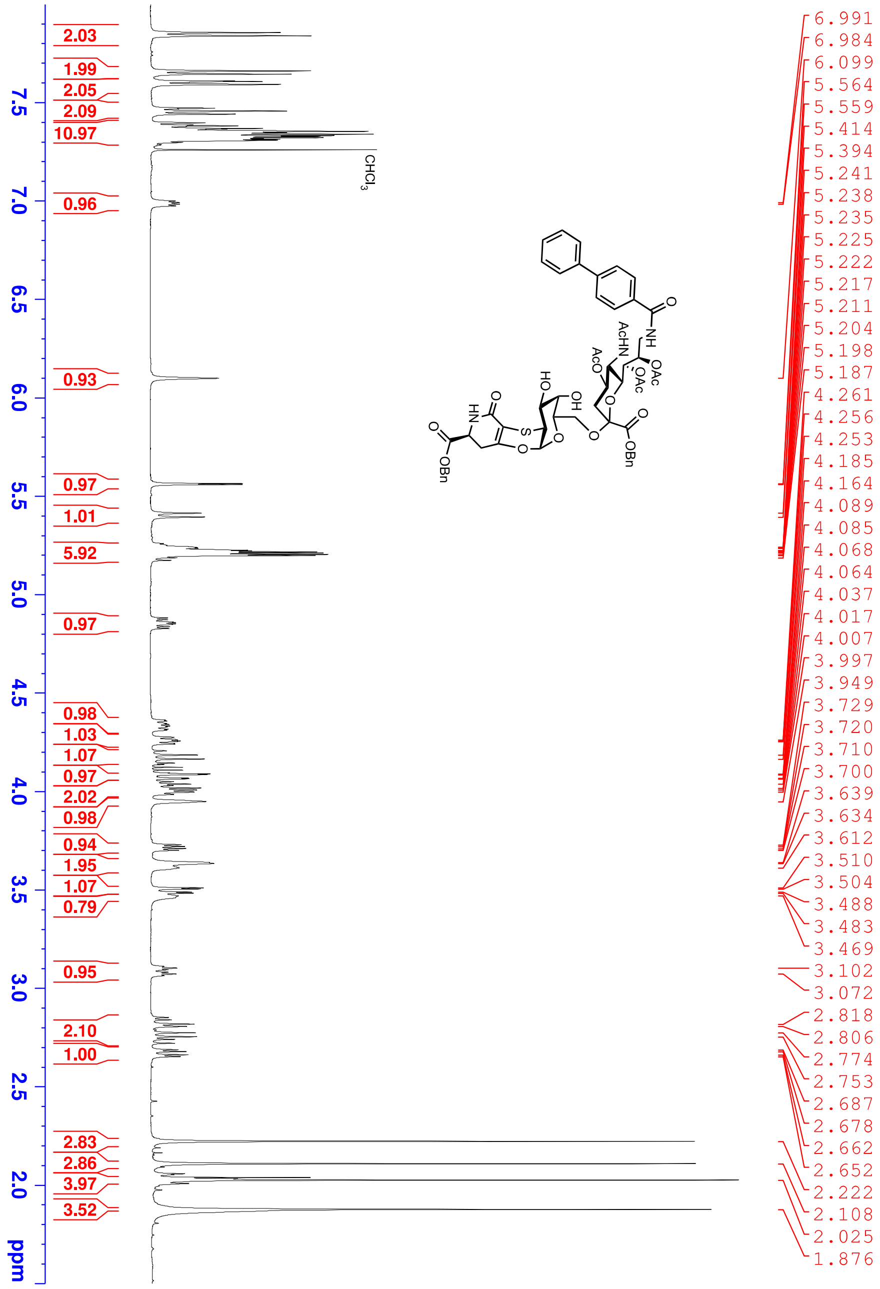

Figure S9. ^{1}H NMR spectrum of **9** (500 MHz, $CDCl_3$).

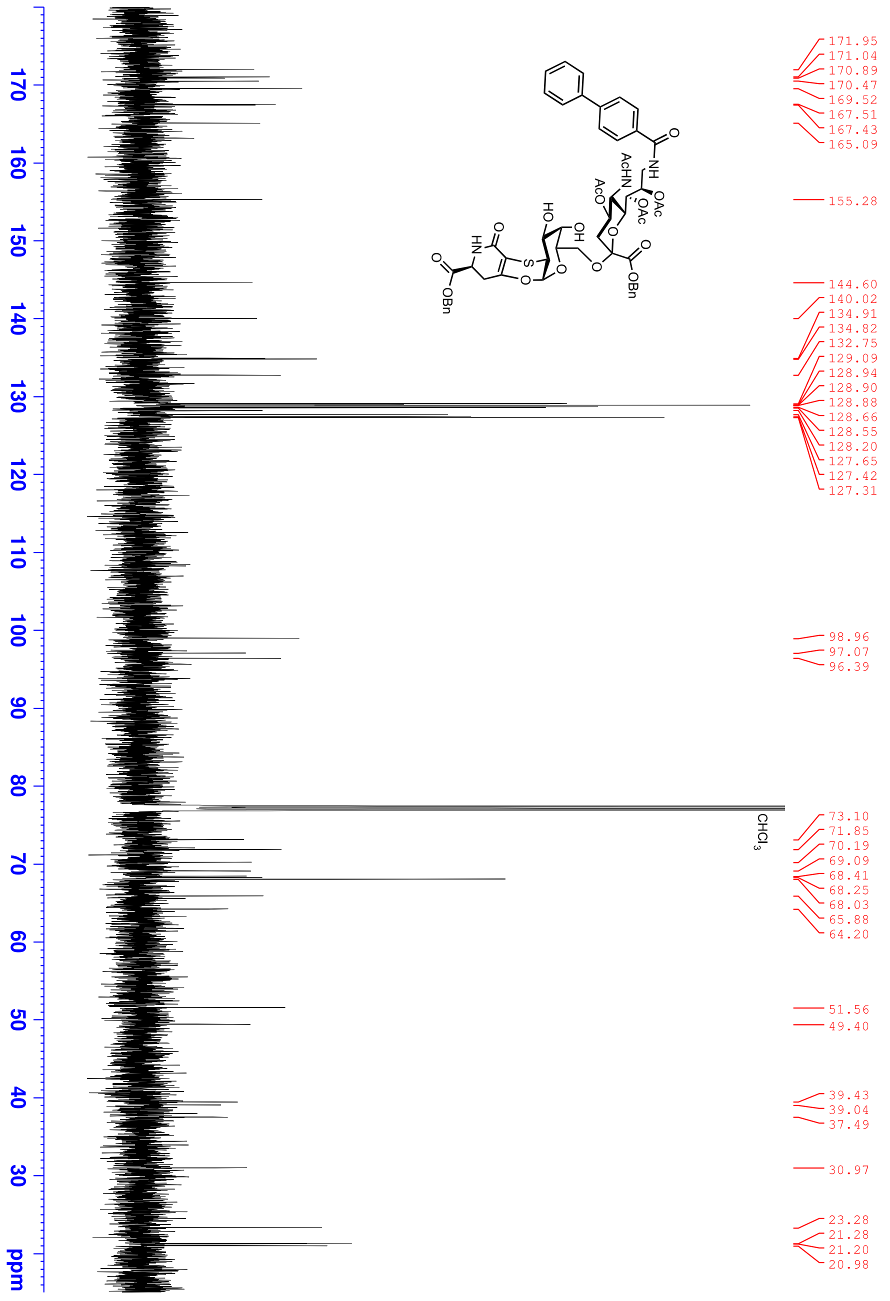

Figure S10. ^{13}C NMR spectrum of **9** (125 MHz, $CDCl_3$).

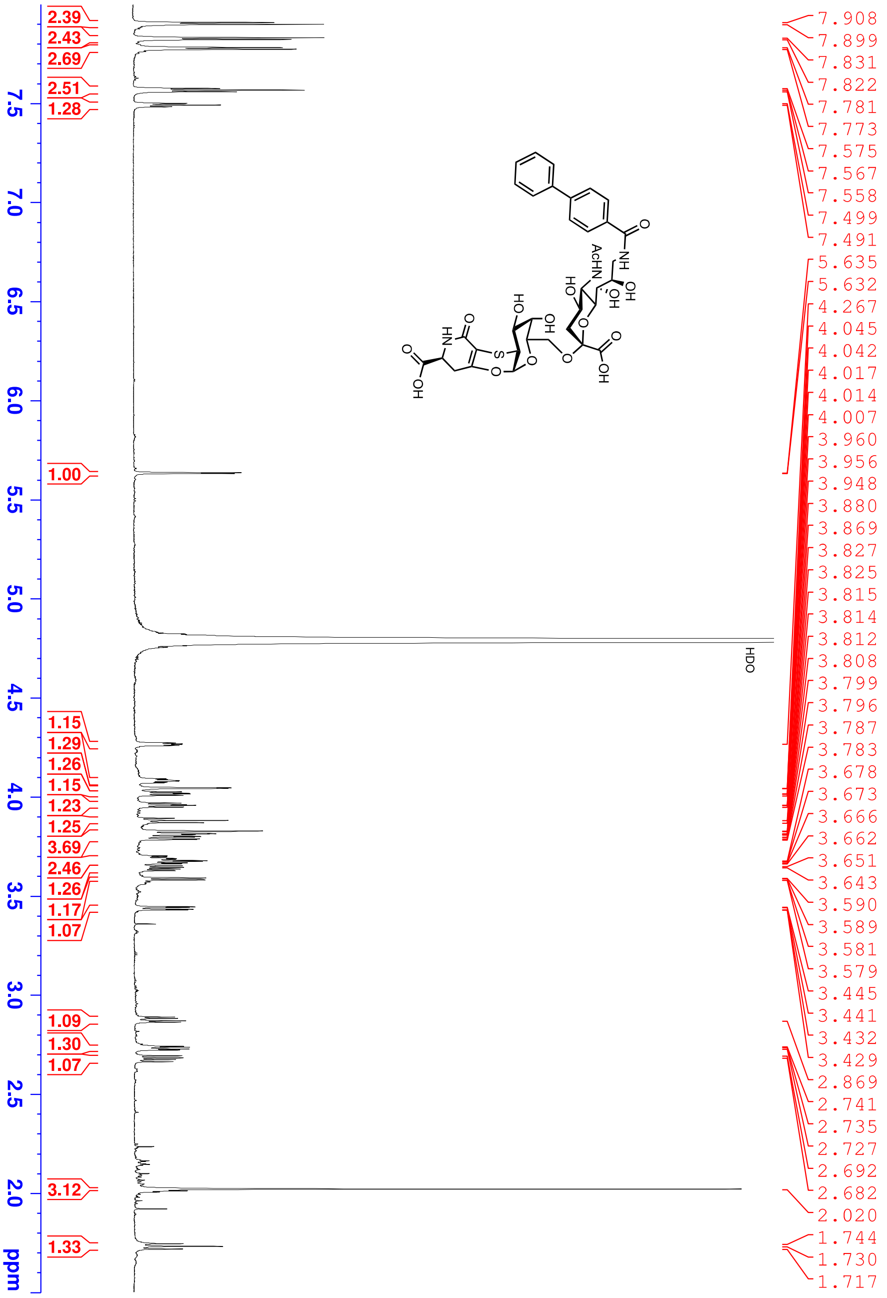

Figure S11. ^{1}H NMR spectrum of **2** (700 MHz, D_2O).

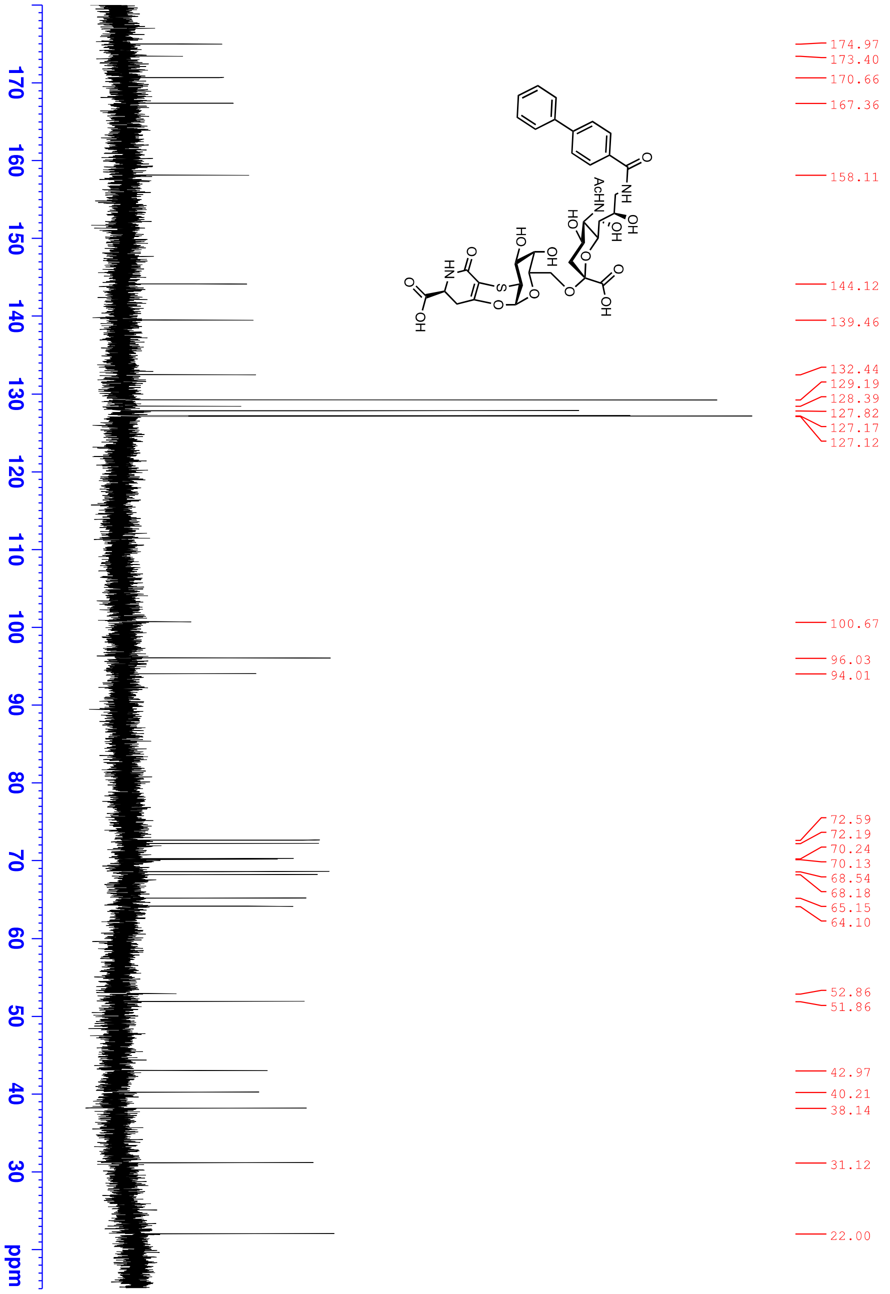

Figure S12. ${}^{13}C$ NMR spectrum of **2** (175 MHz, D_2O).

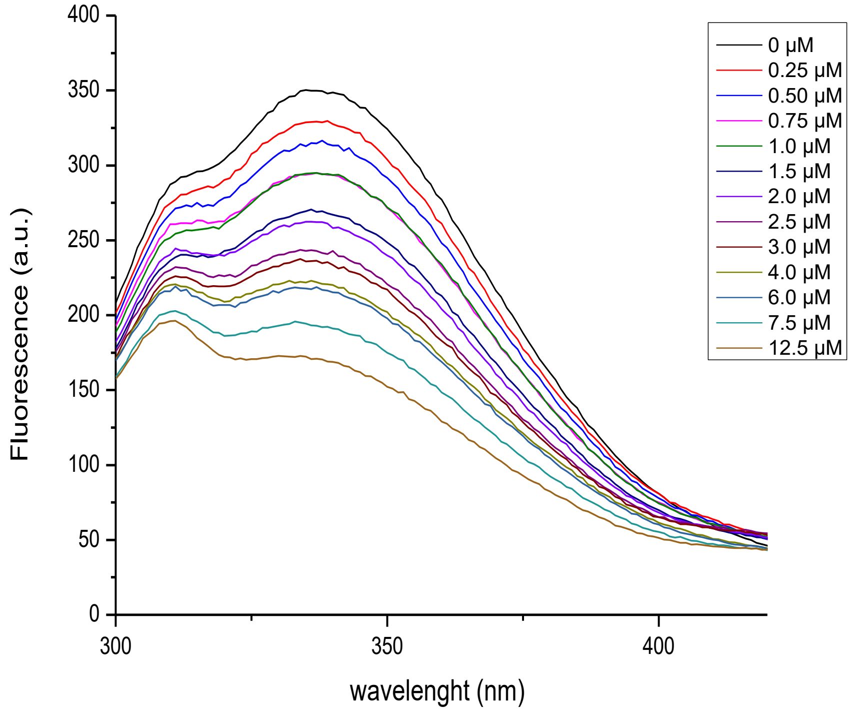

Figure S13. Fluorescence spectra of h-CD22 (black line) in the presence of increasing amounts of **1** (colored lines).

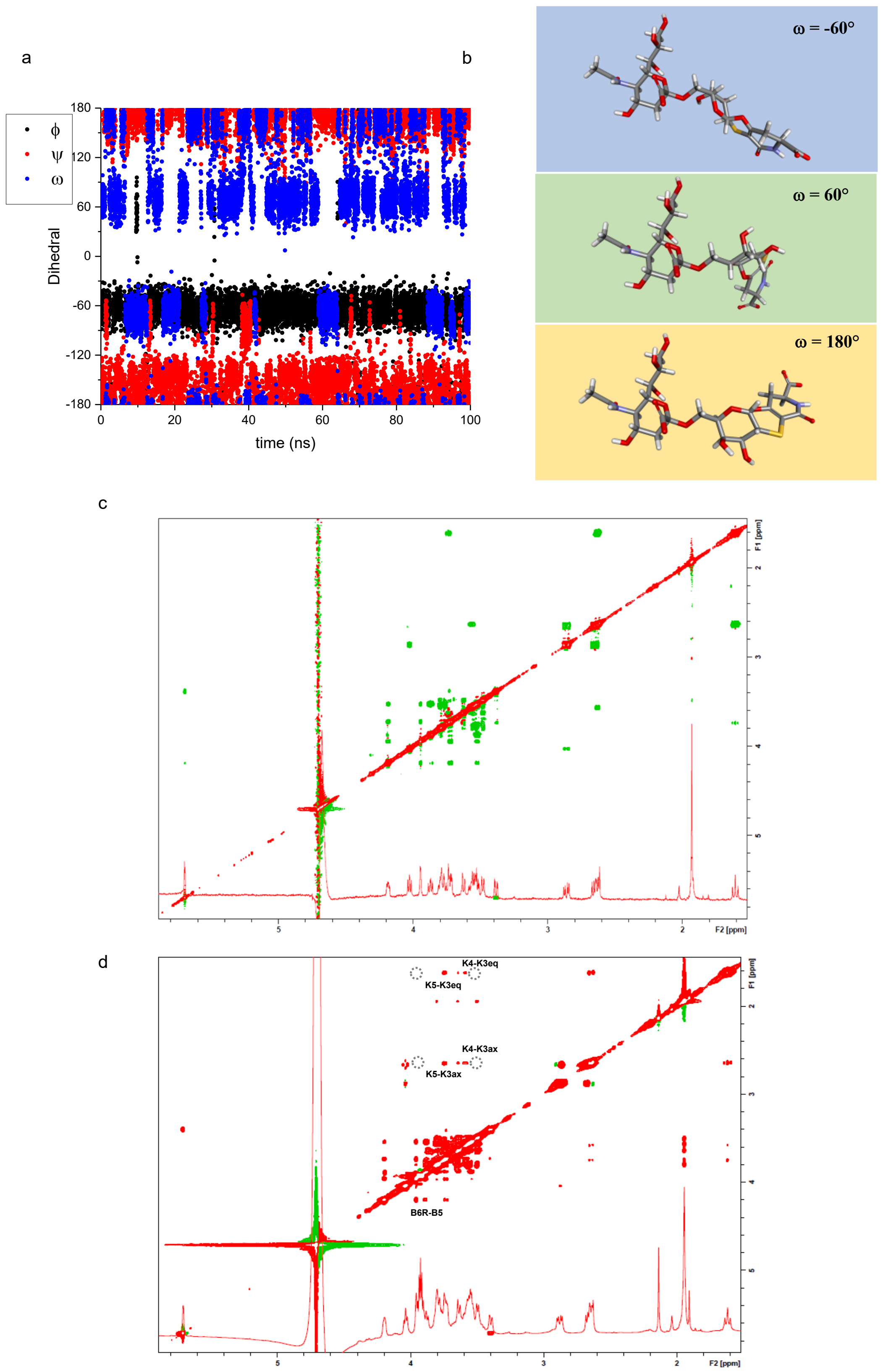

Figure S14. Conformational analysis of 1. a) Dihedral angle fluctuations around the Neu5Ac-Gal linkage along the MD simulation of 1 in the free state. b) 3D representation of the main conformations of 1 according to the three different values of ω torsion. c) T-ROESY (mixing time: 500ms) of 1 in the free state d) tr-NOESY (mixing time: 300ms) of 1:30 h-CD22/ 1 mixture (T=298K)

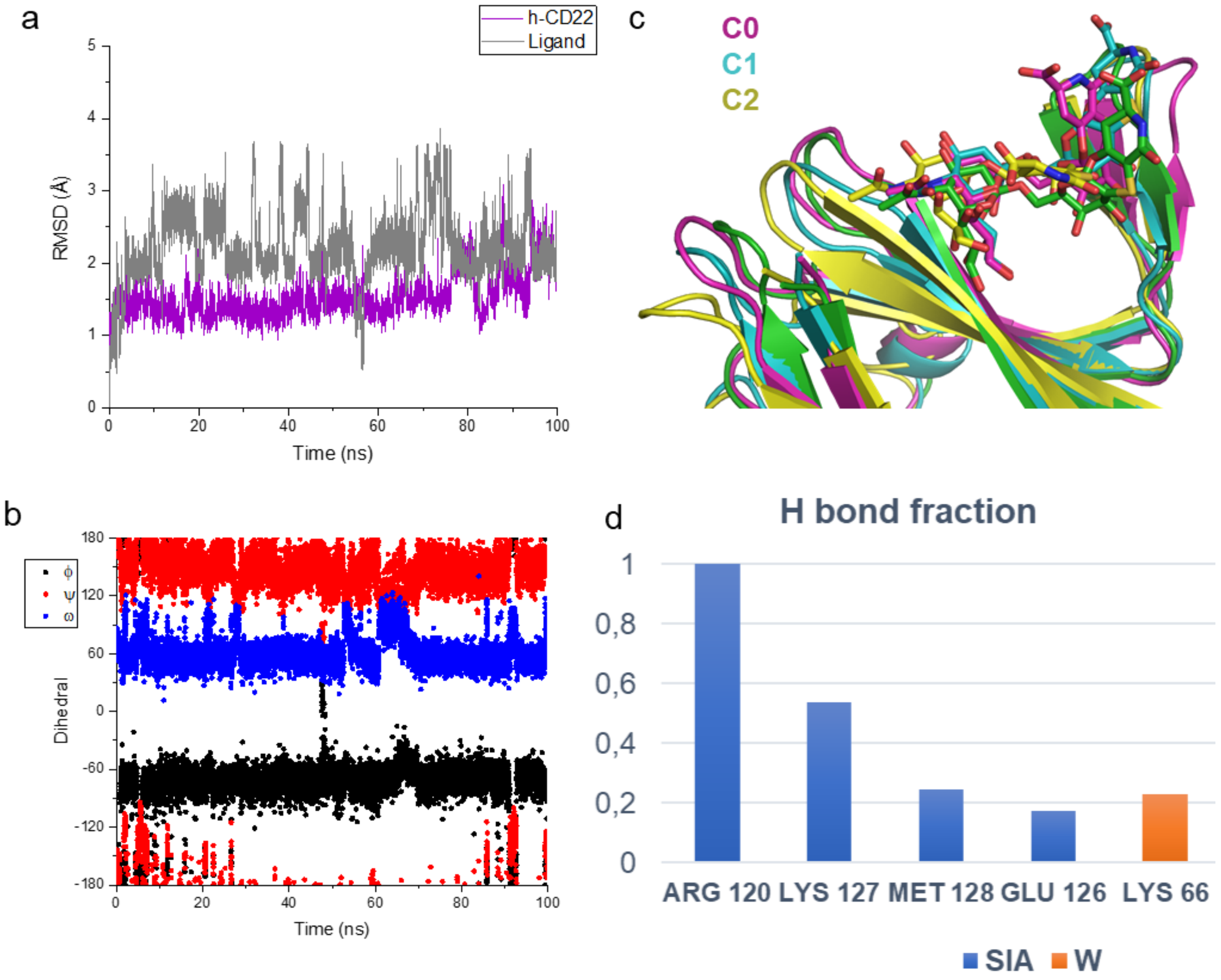

Figure S15. MD simulation analysis h-CD22/1 complex. a) Protein and ligand RMSD. The ligand RMSD was calculated in reference to the protein. b) Superimposition of the three most populated clusters from the MD simulation. Kmean algorithm was considered for clustering. c) Fluctuation of Neu5Ac-Gal dihedral angles of 1 along the MD simulation. d) Protein/ligand H bonds fraction.

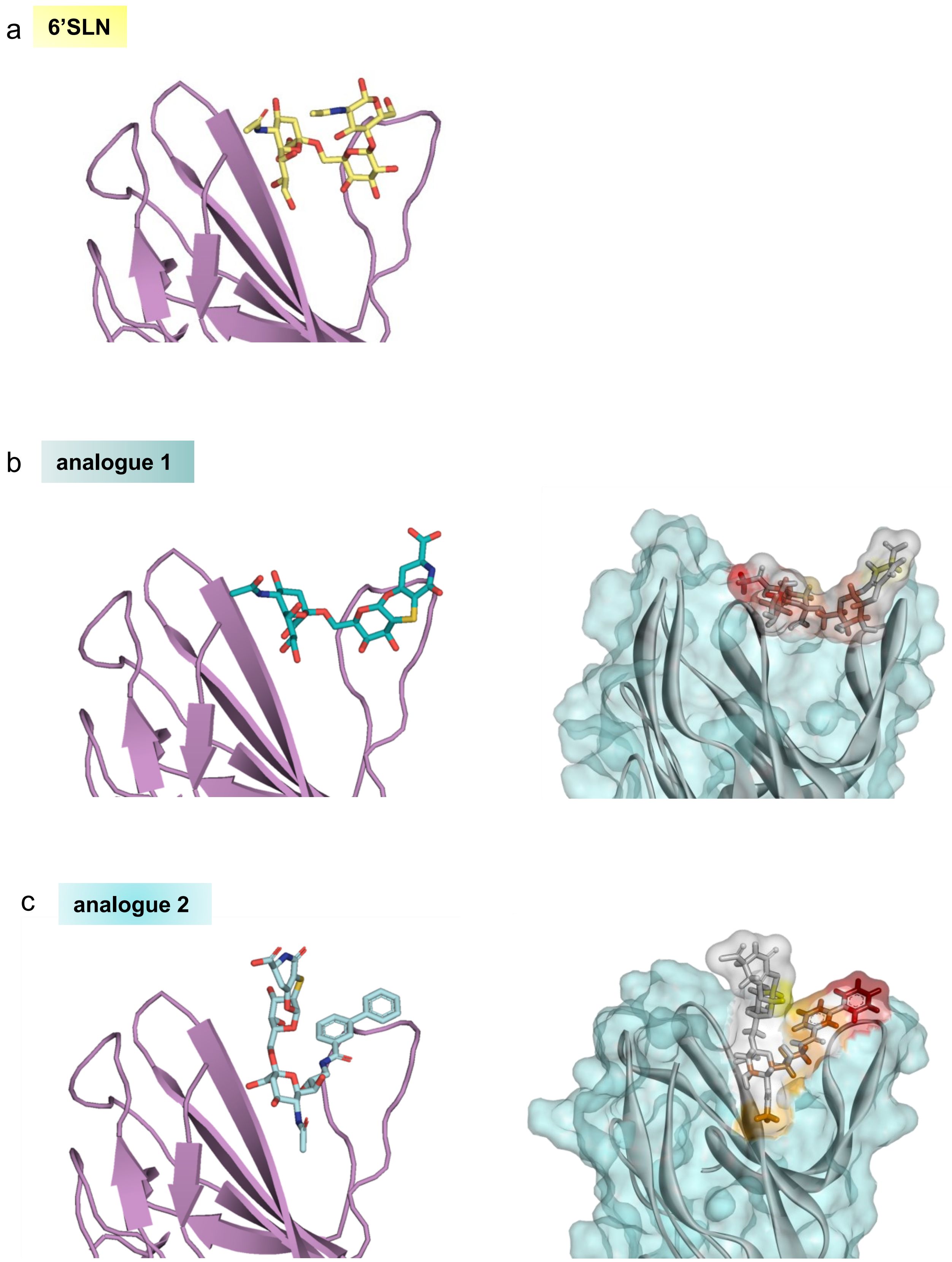

Figure S16. 3D view of the h-CD22/6’SLN, h-CD22/1 and h-CD22/2 complexes. On the right: Representations of the binding pose of 6’SLN (a), **1** (b) and **2** (c) in the binding pocket of h-CD22. **On the left:** 3D view of **h-CD22/1** (b) and **h-CD22/2** (c) complexes in accordance with STD, tr-NOESY and MD data. The ligand surface is colored according to the STD effects.

Experimental Procedures

Protein expression and purification

The plasmids encoding for the three N-terminal Ig-like domains of human CD22 fused to the Fc region of mouse IgG2b was expressed in CHO cell lines and purified as described elsewhere.[1]

Synthesis and characterization of chemical materials

ESI-MS analyses were performed in negative ion mode and were recorded on an LCQ-Fleet Ion Trap equipped with a standard Ionspray interface. HRMS were performed on a Triple-TOF with a resolution of 35000 (FWHM). Chemical shifts are reported in part per million (δ) using the residual solvent line as secondary internal reference. ^{1}H NMR spectra were obtained at 500 MHz and 700 MHz, chemical shifts are reported in δ. ^{13}C NMR spectra were obtained at 125 MHz, chemical shifts are reported in δ. Polarimetry analysis were performed on a Jasco DIP-370.

Synthesis of compound **3**. To a solution of *N*-acetylneuraminic acid (6.00 g, 19.4 mmol) in 15 mL of DMF, cooled to 0 °C, 1,8-diazabicyclo[5.4.0]undec-7-ene (DBU) (4.40 mL, 29.5 mmol) and benzyl bromide (3.40 mL, 28.6 mmol) were added. The solution was stirred at room temperature for 18h and then poured into 1 L of cold CH_2Cl_2. The precipitate was collected by filtration, washed with CH_2Cl_2 (7 x 100 mL) and dried in vacuo, to afford 6.23 g of crude as a white solid. To a solution of crude (2.00 g, 5.20 mmol) in 20 mL of pyridine, cooled to 0 °C, TsCl (6.00 mL, 7.80 mmol) was added and the solution was stirred at room temperature for 18h, then, Ac_2O (16 mL) and a catalytic amount of DMAP were added, and the solution was stirred for 5 h at room temperature. The reaction mixture was diluted with 200 mL of AcOEt and the organic layer washed with 1 M HCl and then with water. The organic phase was then collected, dried over anhydrous Na_2SO_4, filtered and evaporated, to afford 5.02 g of crude as a yellow oil. Crude was purified by flash-cromathogrophy on silica gel (petroleum ether 10% in AcOEt) to afford **3** as a yellow amorphous solid (1.22 g, 40%). ^{1}H NMR (500 MHz, $CDCl_3$, ∂ = 7.26): ∂ 7.75 (d, J = 8.4 Hz, 2H, Ar); 7.38-7.29 (m, 7H, Ar); 5.56 (d, J = 9.7 Hz, N*H*Ac); 5.38 (dd, J_1 = 4.9 Hz, J_2 = 2.2 Hz, 1H, C*H*-7); 5.24 (td, J_1 = 10.9 Hz, J_2 = 4.9 Hz, 1H, C*H*-4); 5.16 (q, 2H, CH_2Ph); 5.03-5.00 (m, 1H, C*H*-6); 4.50 (dd, J_1 = 11.3 Hz, J_2 = 2.8 Hz, 1H, C*H*-9); 4.13-4.03 (m, 3H, C*H*-8, C*H*-5, C*H*-9); 2.51 (dd, J_1 = 13.3 Hz, J_2 = 5.1 Hz, 1H, C*H*-3_{eq}); 2.42 (s, 3H, SO_2Ph*Me*); 2.08 (s, 3H, *Ac*); 2.06 (s, 3H, *Ac*); 2.03 (s, 4H, *Ac*, C*H*-3_{ax}); 2.00 (s, 3H, *Ac*); 1.95 (s, 3H, *Ac*); 1.87 (s, 3H, *Ac*). ^{13}C NMR (125 MHz, $CDCl_3$, ∂ = 77.16): ∂ 171.32; 171.00; 170.58; 170.32; 168.37; 165.60; 144.83; 134.96; 133.10; 129.90; 128.71; 128.63; 128.3; 128.09; 97.53 (*C*-2); 72.75 (*C*H-8); 70.86 (*C*H-6); 68.29 (*C*H-4); 68.00 (*C*H_2Ph); 67.72 (*C*H_2-9); 67.54 (*C*H-7); 49.36 (*C*H-5); 36.02 (*C*H_2-3); 23.26 (SO_2Ph*Me*); 21.75; 21.16; 20.93; 20.78.

Synthesis of compound **4**. To a solution of **3** in 15 mL of anhydrous DMF, NaN_3 (600 mg, 9.36 mmol) was added and the mixture was heated to 70 °C and stirred for 5h. Then, a second amount of NaN_3 (300 mg, 3.18 mmol) was added and the mixture was stirred at 70 °C for 1 h. The reaction mixture was diluted with 400 mL of AcOEt and the organic phase washed with brine. The organic layer was collected, dried over anhydrous Na_2SO_4, filtered and evaporated, to afford 1.10 g of crude as a brown solid. The crude was purified by flash-chromatography on silica gel (AcOEt) to afford pure **4** as an amorphous solid (450 mg, 50%). ^{1}H NMR (500 MHz, $CDCl_3$, ∂ = 7.26): ∂ 7.38-7.34 (m, 5H, *Ar*); 5.50 (d, J = 9.8 Hz, 1H, N*H*Ac); 5.37 (dd, J_1 = 3.4 Hz, J_2 = 2.5 Hz, 1H, C*H*-7); 5.26-5.14 (m, 3H, C*H*-4, CH_2Ph); 4.87 (dt, J_1 = 7.9 Hz, J_2 = 3.2 Hz, 1H, C*H*-8); 4.15 (q, J = 10.2 Hz, 1H, C*H*-6); 4.06 (dd, J_1 = 10.6 Hz, J_2 = 2.6 Hz, 1H, C*H*-5); 3.77 (dd, J_1 = 13.5 Hz, J_2 = 2.7 Hz, 1H, C*H*-9); 3.30 (dd,

J_1 = 13.5 Hz, J_2 = 8.0 Hz, 1H, C*H*-9); 2.51 (dd, J_1 = 13.3 Hz, J_2 = 5.0 Hz, 1H, C*H*-3$_{eq}$); 2.14 (s, 3H, *Ac*); 2.11 (s, 3H, *Ac*); 2.05 (s, 3H, *Ac*); 2,04 (bs, 1H, C*H*-3$_{ax}$); 2.02 (s, 3H, *Ac*); 1.87 (s, 3H, *Ac*). ^{13}C NMR (125 MHz, $CDCl_3$, ∂ = 77.16): ∂ 171.11; 170.92; 170.51; 170.45; 168.53; 165.81; 134.87; 128.75; 128.48; 97.39 (*C*-2); 73.88 (*C*H-8); 73.46 (*C*H-5); 68.46 (*C*H-7); 68.35 (*C*H-4); 68.21 (*C*H_2Ph); 50.16 (*C*H_2-9); 49.26 (*C*H-6); 36.24 (*C*H_2-3); 23.36; 21.02; 20.95; 20.88; 20.80.

Synthesis of compound **5**. To a solution of **4** (790 mg, 1.33 mmol) in 15 mL of anhydrous CH_2Cl_2, cooled to 0 °C, PhSH (160 µL, 1,55 mmol) and $BF_3 \cdot Et_2O$ (402 µL, 3.26 mmol) were added. The solution was stirred at room temperature for 18h. The solution was diluted with 100 mL of CH_2Cl_2 and the organic layer washed with a saturated solution of $NaHCO_3$, dried over anhydrous Na_2SO_4, filtered, and evaporated, to give 820 mg of crude. Crude was purified by flash-chromatography on silica gel (petroleum ether 20% in AcOEt), to obtain **5** as a white amorphous solid (780 mg, 92%). ^{1}H NMR (500 MHz, $CDCl_3$, ∂ = 7.26): ∂ 7.36-7.24 (m, 10H, *Ar*); 5.89 (d, *J* = 9.8 Hz, 1H, N*H*Ac); 5.43 (t, *J* = 2.0 Hz, 1H, C*H*-7); 5.39 (td, , J_1 = 11.0 Hz, J_2 = 4.7 Hz, 1H, C*H*-4); 5.05 (dd, , J_1 = 63.38 Hz, J_2 = 12.2 Hz, 2H, C*H_2*Ph); 4.67 (dt, J_1 = 9.0 Hz, J_2 = 1.9 Hz, 1H, C*H*-8); 4.57 (dd, J_1 = 10.6 Hz, J_2 = 2.4 Hz, 1H, C*H*-6); 4.16-4.13 (m, 1H, C*H*-5); 3.52 (dd, J_1 = 13.6 Hz, J_2 = 1.4 Hz, 1H, C*H*-9); 3.20 (dd, J_1 = 13.6 Hz, J_2 = 9.5 Hz, 1H, C*H*-9); 2.68 (dd, J_1 = 13.8 Hz, J_2 = 4.8 Hz, 1H, C*H*-3$_{eq}$); 2.14 (bs, 1H, C*H*-3$_{ax}$); 2.09 (s, 3H, *Ac*); 2.06 (s, 3H, *Ac*); 2.03 (s, 3H, *Ac*); 1.87 (s, 3H, *Ac*). ^{13}C NMR (125 MHz, $CDCl_3$, ∂ = 77.16): ∂ 171.28; 171.05; 170.49; 170.29; 167.55; 135.88; 130.11; 129.47; 128.72; 128.69; 88.66 (*C*-2); 74.78 (*C*H-8); 73.24 (*C*H-6); 69.14 (*C*H-4); 68.88 (*C*H-7); 67.88 (*C*H_2Ph); 49.74 (*C*H-9); 49.37 (*C*H-5); 37.64 (*C*H_2-3); 23.18; 21.10; 20.96; 20.82.

Synthesis of compound **7**. Under nitrogen atmosphere, to a solution of **5** (780 mg, 1.21 mmol) and **6**[1] (1.08 g, 4.90 mmol) in 80 mL of CH_2Cl_2, PPh_3 was added (650 mg, 2.87 mmol). The solution was stirred for 48h and then the solvent was removed, to obtain 2.34 g of crude as a white solid. Crude was purified by flash-chromatography on silica gel (petroleum ether 20% in AcOEt), to obtain pure **7** as an amorphous white solid (278 mg, 30%). ^{1}H NMR (500 MHz, $CDCl_3$, ∂ = 7.26): ∂ 7.77 (d, *J* = 8.3 Hz, 2H, *Ar*); 7.64-7.62 (m, 4H, *Ar*); 7.48 (t, *J* = 8.0 Hz, 2H, *Ar*); 7.41-7.38 (m, 1H, *Ar*); 7.32 (d, *J* = 1.5 Hz, 5H, *Ar*); 7.27-7.25 (m, 2H, *Ar*); 7.16-7.13 (m, , 3H, *Ar*); 6.77 (t, *J* = 6.4 Hz, 1H, N*H*); 5.69 (d, *J* = 10.3 Hz, 1H, N*H*Ac); 5.45-5.40 (m, 2H, C*H*-4, C*H*-7); 5.11 (bs, 2H, C*H_2*Ph); 4.92 (q, *J* = 4.0 Hz, 1H, C*H*-8); 4.67 (dd, J_1 = 10.6 Hz, J_2 = 2.6 Hz, 1H, C*H*-6); 4.22 (q, *J* = 10.4 Hz, 1H, C*H*-5); 3.94 (ddd, J_1 = 15.0 Hz, J_2 = 6.8 Hz, J_3 = 3.3 Hz, 1H, C*H*-9); 3.31, (dt, J_1 = 15.0 Hz, J_2 = 5.4 Hz, 1H, C*H*-9); 2.67 (dd, J_1 = 13.8 Hz, J_2 = 4.8 Hz, 1H, C*H*-3$_{eq}$); 2.21-2.15 (m, 1H, C*H*-3$_{ax}$); 2.13 (s, 3H, *Ac*); 2.03 (s, 3H, *Ac*); 2.02 (s, 3H, *Ac*); 1.89 (s, 3H, *Ac*). ^{13}C NMR (125 MHz, $CDCl_3$, ∂ = 77.16): ∂ 171.33; 161.15; 171.07; 171.01; 167.76; 167.06; 144.21; 140.23; 136.13; 134.81; 132.26; 132.18; 132.12; 129.96; 129.37; 129.08; 128.80; 128.75; 128.71; 128.61; 128.11; 127.78; 127.30; 127.15; 88.62 (*C*-2); 73.31 (*C*H-8); 72.79 (*C*H-6); 69.23 (*C*H-4); 69.16 (*C*H-7); 67.95 (*C*H_2Ph); 49.62 (*C*H-5); 39.31 (*C*H-9); 37.36 (*C*H_2-3); 23.27; 21.26; 21.07; 21.00.

Synthesis of compound **9**. Under nitrogen atmosphere, to a solution of **7** (65 mg, 0.140 mmol) and **8**[2] (278 mg, 0.350 mmol) in 1.5 mL of an anhydrous mixture of CH_3CN/CH_2Cl_2 (10:1), cooled at -40 °C, NIS (132 mg, 0.580 mmol) and TfOH (25 µL, 0.280 mmol) were added. The reaction mixture was stirred at -40 °C for 4 h, then Et_3N was added (150 µL) and the mixture was let slowly return to room temperature. The reaction mixture was diluted with 20 mL of CH_2Cl_2 and the organic layer washed with a 10% $Na_2S_2O_3$ solution. The organic layer was collected, dried over anhydrous Na_2SO_4, filtered and solvent evaporated, to obtain 275 mg of crude. Crude was purified by flash-chromatography on silica gel (AcOEt), to obtain 175 mg of a partially purified mixture. The mixture

was dissolved in 10 mL of AcOH 80% and heated at 45 °C for 18h. The solvent was removed and crude purified by flash-chromatography on silica gel (MeOH 5% in AcOEt), to obtain pure **9** as an amorphous white solid (60 mg, 40%) ^{1}H NMR (500 MHz, $CDCl_3$, ∂ = 7.26): ∂ 7.85 (d, *J* = 8.4 Hz, 2H, *Ar*); 7.65 (d, *J* = 8.4 Hz, 2H, *Ar*); 7.61-7.59 (m, 2H, *Ar*); 7.46 (t, *J* = 7.3 Hz, 2H, *Ar*); 7.39-7.31 (m, 11H, *Ar*); 6.99 (dd, , J_1 = 8.4 Hz, J_2 = 3.9 Hz, 1H, N*H*); 6.10 (s, 1H, N*H*); 5.56 (d, *J* = 2.56 Hz, 1H, C*H*-1); 5.40 (d, *J* = 10.0 Hz, 1H, N*H*Ac); 5.25-5.17 (m, 6H, C*H*-7', C*H*-8', CH_2Ph x 2); 4.88-4.83 (m, 1H, C*H*-4'); 4.34 (ddd, J_1 = 14.9 Hz, J_2 = 8.5 Hz, J_3 = 2.4 Hz, 1H, C*H*-9'); 4.26 (ddd, J_1 = 10.1 Hz, J_2 = 6.0 Hz, J_3 = 1.2 Hz, 1H, C*H*-b); 4.17 (q, 1H, *J* = 10.4 Hz, C*H*-5'); 4.09-4.06 (m, 1H, C*H*-6); 4.05-4.00 (m, 2H, C*H*-4, C*H*-5); 3.95 (bs, 1H, C*H*-6'); 3.72 (dd, J_1 = 9.5 Hz, J_2 = 4.7 Hz, 1H, C*H*-6); 3.64-3.61 (m, 2H, C*H*-3, O*H*); 3.47 (dd, J_1 = 10.8 Hz, J_2 = 2.5 Hz, 1H, C*H*-2); 3.47 (bs, 1H, O*H*); 3.09 (dt, J_1 = 14.9 Hz, J_2 = 4.6 Hz, 1H, C*H*-9'); 2.85-2.72 (m, 2H, CH_2-a); 2.67 (dd, J_1 = 12.7 Hz, J_2 = 4.9 Hz, 1H, C*H*-3'$_{eq}$); 2.22 (s, 3H, *Ac*); 2.10 (s, 3H, *Ac*); 2.02 (s, 4H, *Ac*, C*H*-3'$_{ax}$); 1.87 (s, 3H, *Ac*). ^{13}C NMR (125 MHz, $CDCl_3$, ∂ = 77.16): ∂ 171.95; 171.03; 170.89; 170.47; 169.52; 167.51; 167.43; 165.09; 155.28; 144.60; 140.02; 134.91; 134.82; 132.75; 129.09; 128.94; 128.90; 128.88; 128.66; 128.55; 128.20; 127.65; 127.41:127.31; 98.96 (*C*-2'); 97.07; 96.39 (C*H*-1); 73.10 (C*H*-4); 71.85 (C*H*-5); 70.19 (C*H*-5'); 69.09 (C*H*-7'); 68.41 (C*H*-4'); 68.25 (C*H*-8'); 68.03 (C*H*-6', *C*H_2Ph x 2); 65.88 (*C*H-3); 64.20 (*C*H-2); 51.56 (*C*H-b); 49.40 (*C*H_2-9'); 39.43 (*C*H_2-6); 37.49 (*C*H_2-3'); 30.97 (*C*H_2-a); 23.28; 21.27; 21.20; 20.98.

Synthesis of sialo-derivative **2**. To a solution of **9** (20 mg, 0.017 mmol) in 4 mL of MeOH, Pd/C was added (24 mg). The suspension was stirred for 73h under hydrogen atmosphere, then, the suspension was filtered on an HPLC filter and the solution evaporated to afford 7 mg of crude. Crude was dissolved in 1 mL of NH_3 4M in MeOH and the solution was stirred at room temperature for 120h. The solution was evaporated and the crude was suspended in 1 mL of Et_2O/MeOH (6:4) and centrifugated (5' at 3000 rpm), the supernatant was eliminated and the procedure repeated 10 times. The purified product was dried under high vacuum to obtain pure **2** (4 mg, 30%). ^{1}H NMR (700 MHz, D_2O, ∂ = 4.79): ∂ 7.90 (d, *J* = 8.2 Hz, 2H, *Ar*); 7.83 (d, *J* = 8.2 Hz, 2H, *Ar*); 7.78 (d, *J* = 8.2 Hz, 2H, *Ar*); 7.57 (t, *J* = 7.6 Hz, 2H, *Ar*); 7.49 (t, *J* = 7.3 Hz, 1H, *Ar*); 5.63 (d, *J* = 8.2 Hz, 2H, C*H*-1); 4.26 (dd, J_1 = 6.9 Hz, J_2 = 3.9 Hz, C*H*-8'); 4.09-4.07 (m, 1H, C*H*-5); 4.04 (d, *J* = 2.8 Hz, 1H, C*H*-7'); 4.02 (dd, J_1 = 9.5 Hz, J_2 = 6.7 Hz, 1H, C*H*-9'); 3.96 (dd, J_1 = 10.7 Hz, J_2 = 7.7 Hz, 1H, C*H*-a); 3.88 (t, *J* = 9.9 Hz, 1H, C*H*-5'); 3.82-3.78 (m, 3H, C*H*-3, C*H*-6, C*H*-6'); 3.70-3.63 (m, 3H, C*H*-4', C*H*-4, C*H*-9'); 3.59 (dd, J_1 = 8.7 Hz, J_2 = 1.3 Hz, 1H, C*H*-6); 3.44 (dd, J_1 = 11.4 Hz, J_2 = 2.9 Hz, 1H, C*H*-2); 2.87 (dd, J_1 = 16.8 Hz, J_2 = 6.6 Hz, 1H, C*H*-b); 2.73 (dd J_1 = 12.2 Hz, J_2 = 4.6 Hz, 1H, C*H*-3$_{eq}$); 2.68 (dd, J_1 = 17.2 Hz, J_2 = 9.9 Hz, 1H, C*H*-b); 2.02 (s, 3H, *Ac*); 1.73 (t, *J* = 12.0 Hz, 1H, C*H*-3'$_{ax}$). ^{13}C NMR (175 MHz, D_2O, ∂): ∂ 174.97; 173.40; 170.66; 167.36; 158,11; 144.12; 139.46; 132.44; 129.19; 128.39; 127.82; 127.17; 127.12; 100.67; 96.02 (*C*H-1); 94.01; 72.59 (*C*H-3); 72.19 (*C*H-8'); 70.23 (*C*H-5); 70.13 (*C*H-4); 68.54 (*C*H-7'); 68.18 (*C*H-b); 65.15 (*C*H-4'); 64.10 (*C*H-6'); 52.86 (*C*H-9'); 51.86 (*C*H_2-5'); 42.97 (*C*H_2-6); 40.21 (*C*H_2-3'); 38.13 (*C*H-2); 31.11(*C*H-a); 22.00. ESI-MS m/z (%): 400.83 (100%) $[M-2H/]^{2-}$, HRMS (m/z): $[M-2H/]^{-}$, calcd. for $C_{36}H_{40}O_{16}$ N_3S, 802.21348, found 802.21155; $[\alpha]_D^{21°C}$ = +6 (0.5 mg/mL in H_2O).

Surface Plasmon Resonance (SPR) analysis

The SPR measurements were performed on a Biacore X100 instrument (Cytiva, Global Life Sciences Solutions, Marlborough, USA). Protein A (10600-P07E, Sino Biological Inc., Beijing, China) was immobilized on both flow cells (FC1 and FC2) of a gold sensor chip (Cytiva) reaching ~1200 response units (RU) by using a protein solution of 30 mg L^{-1} in 10 mM acetate buffer pH 5.0 injected over the gold surface for 10 min at a flow rate of 10 μL min^{-1}. CD22 protein was captured on the

sensor chip injecting 40 mg L^{-1} of CD22 in 10 mM acetate buffer pH 5 over FC2 at a flow rate of 5 µL min^{-1} for 3 min, and using FC1 as the reference surface; Both flow cells were equilibrated with HBS-EP buffer overnight at a flow rate of 5 µL min^{-1}, achieving for FC2 ~5000 RU. Twofold dilution series of the sialic acid analogues, the analytes, were freshly prepared in HBS-EP running buffer. All binding experiments were performed at 25 °C at a flow rate of 30 µL min^{-1}. The samples were injected for 1 min followed by 1 min dissociation. Each sample concentration was measured in triplicate. Double referencing was applied to correct for bulk effects and other systematic artifacts (subtraction of reference surface and blank injections). Data processing was performed by using the Biacore X100 evaluation software. The dissociation constant (K_D) for the analyte interaction with CD22 was determined according to the common 1:1 binding model described by the equation: $RU = RU_{max} \times \frac{[Analyte]}{[Analyte]+K_D}$, where RU_{max} is the maximum SPR response, and K_D corresponds to the analyte concentration that gives half of the maximum SPR response, *i.e.*, $RU_{max}/2$.[2-3]

Fluorescence analysis

The experiments of steady-state fluorescence spectroscopy have been carried out on a Fluoromax-4 spectrofluorometer (Horiba, Edison, NJ, USA) at the fixed temperature of 5 °C; an excitation at 280 nm was used and emission spectra were recorded in the range of 290–500 nm. The slit widths were fixed at 4 nm for the excitation and 5 nm for the emission wavelength. A quartz cuvette with a path length of 1 cm and a chamber volume of 1 mL was used under constant stirring. 0.9 mL of CD22 solution at fixed concentration of 0.25 µM was titrated by adding small volumes (1–20 µL of a ligand stock solution of 500 µM) of analogue **1**. The PBS buffer at pH 7.4 was used for all solutions. The optical density of the solution at the excitation wavelength was kept less than 0.05.

The data were analyzed by non-linear regression with O*ne Site- Specific Binding* model for the determination of the dissociation constant (K_D) as implemented in in OriginPro 2016, according to the following equation. [4]

$$Y = \frac{B_{max} * X}{K_D + X}$$

where X stands for the ligand concentration, Y is the change of the fluorescence intensity at the maximum wavelength, B_{max} represents the maximum specific binding and K_D is the equilibrium dissociation constant.

NMR

NMR spectra were acquired on a Bruker 600-MHz Avance Neo instrument fitted with a cryo probe. NMR samples were dissolved in 50 mM deuterate phosphate buffer (NaCl 140 mM, Na_2HPO_4 10 mM, KCl 3 mM, pH 7.4) and the [D4](trimethylsilyl)propionic acid, sodium salt (TSP, 10 uM) was used as internal reference to calibrate all the spectra. Data acquisition and processing were analyzed using TOPSPIN 3.2 software. The chemical shifts of the glycan ligands were assigned by ^{1}H, COSY, TOCSY, NOESY and HSQC experiments. Homonuclear 2D ^{1}H-^{1}H NOESY experiments were carried out by using data sets of 2048x512 points and mixing times of 300 ms.

^{1}H NMR spectra were registered by using 16 k and 32 k data points and zero-filled up to 64 k data points prior to processing. The 2D homonuclear spectra were recorded with data sets of 4096x512 (t1 x t2) points and the data matrix processed with zero-filling in the F1 dimension up to 4096x2048 points. In order to improve the resolution, a cosine-bell function was used before Fourier transformation in both dimensions. Heteronuclear single quantum coherence (HSQC) experiments were carried out by using the sequence "hsqcedetgpsisp" from the Bruker library, setting data points of 2048x256.

The STD NMR spectra were acquired with a number of 64 scans, in addition to 32 scans to allow the sample to come to equilibrium, and 64K data points. A protein:ligand ratio of 1:100 and a saturation time of 2 s were used with the on-resonance pulse at 6.5 ppm and the off-resonance at 40ppm. By using these conditions, no STD signals were observed in the control STD NMR spectrum of the ligand alone. A train of 50ms (field strength of 21 Hz) Gaussian shaped pulse with an attenuation of 60db has been used to saturate the protein.
The epitope mapping of ligands **1** and **2** was achieved by the calculation of the ratio $(I_0 - I_{sat})/I_0$, where $(I_0 - I_{sat})$ is the intensity of the signal in the STD NMR spectrum and I_0 is the peak intensity referred to the unsaturated reference spectrum (off-resonance).

Docking

Preparation of the macromolecules.
The crystal structure of h-CD22 extracellular domain was used for docking purposes (PDB ID:5VKM). The structure was submitted to 100000 steps of steepest descent minimization with OPLS3 force field using MacroModel [5] before being used for docking calculations.

Building of ligands. The 3D coordinates of analogues 1 and 2 were built using Maestro program.[6] The ligands geometries were optimized by 100000 step of steepest descent minimization with OPLS3 force field using Macro Model.[5] Ligands were prepared for docking calculations using AutoDockTools setting all rotatable bonds free to move during the calculations except for the glycosidic bonds.

Docking calculations. Docking calculations of all compounds were performed using AutoDock 4.2.2.[7] Analysis of the docking poses was performed with AutoDockTools. The docking protocol was validated by carrying out the docking of CD22 crystallographic structure in complex with Neu5Ac-α-(2-6)-Gal ligand (PDB ID: 5VKM). The 3D structure of Neu5Ac-α-(2-6)-Gal was extracted from the crystallographic structure of CD22.The grid point spacing was set at 0.375 Å, and a hexahedral box was built with x, y, z dimensions: 64 Å, 46 Å, 56 Å centered in the centroid position among the binding site CD22 residues. A total of 200 runs using Lamarckian Genetic algorithm was performed, with a population size of 100, and 250000 energy evaluations. After docking, the 200 solutions were clustered in groups with root-mean-square deviation less than 1.0 Å. The clusters were ranked according to the lowest energy representative of each cluster.

MD simulations
Molecular dynamics calculations were performed within the AMBER 18 software package in explicit water using the following forcefields: Glycam06j-1 for the glycans, FF14SB for the proteins and Gaff for organic moieties.[8]
All the oligosaccharides were built up and minimized by using Maestro package and the carbohydrate builder utility of the glycam website (www.glycam.com),[9] and then the torsional angles were set to the values obtained through the molecular mechanics calculations. For the protein preparation, missing hydrogen atoms were added, and protonation state of ionizable groups and cap termini were computed by using Maestro Protein Preparation Wizard.[10] For the preparation of analogue **1**, xleap antechamber [11] and parmchk2 modules implemented in AMBER were used in order to parametrize the molecule.
The input files were generated using the tleap modules of the AMBER package, the minimization steps were performed using Sander module and molecular dynamic calculations were performed using the PMEMD module.

The corresponding molecules (free and bound analogue **1**) were positioned within an octahedral box of TIP3P water of the proper size and the remote interactions were calculated using a cut off of 10 Angstroms and Counterions were added to neutralize the system. After the preparation of the input files, an energy minimization process was performed to refine the initial structure. The calculations employed SHAKE for the C-H bonds and 1 fs of integration step. Periodic boundary conditions were applied, as well as the smooth particle mesh Ewald method to represent the electrostatic interactions, with a grid space of 1 Å. The system was minimized, at first, holding the solute fixed, while a second minimization was performed on the entire system. Furthermore, the whole system was slowly heated from 0 to 300 K using a weak restrain on the solute and then, the system was equilibrated at 300 K using constant pressure and removing the restrains on the solute. The system coordinates were saved and used for the 100ns simulations using the PMEMD module implemented in AMBER. Coordinate trajectories were recorded each 2 ps throughout production runs, yielding an ensemble of 10000 structures for each complex, which were finally analyzed. The stability of energy, pressure, temperature and other thermodynamic parameters were monitored along the trajectory and then, RMSD, torsions, clusters distances and hydrogen bonds were extracted. Cpptraj[12] (Roe, D. R., & Cheatham, T. E., 3rd; 2013). was the utility used for analyzing and processing trajectories and coordinate files created from the MD simulations. VMD software was used to visualize the trajectory.[13]

References

[1] C. Di Carluccio, R.E. Forgione, M. Montefiori, M. Civera, S. Sattin, G. Smaldone, K. Fukase, Y. Manabe, P.R. Crocker, A. Molinaro, R. Marchetti, A. Silipo, *iScience* **2020**, *24,* 101998.

[2] F. Torrini, P. Palladino, A. Brittoli, V. Baldoneschi, M. Minunni, S. Scarano, *Anal. Bioanal. Chem.* **2019**, *411,* 7709-7716.

[3] F. Torrini, P. Palladino, V. Baldoneschi, S. Scarano, M. Minunni, *Anal. Chim. Acta* **2021**, *1161*, 338481.

[4] P. Sindrewicz, X. Li, E.A. Yates, J.E. Turnbull, L.Y. Lian, L.G. Yu. *Sci Rep.* **2019**, *14, 9,* 11851.

[5] MacroModel, Schrödinger, LLC, New York, NY (USA), **2020**.

[6] Maestro, Schrödinger, LLC, New York, NY (USA), **2021**.

[7] G.M. Morris, R. Huey, W. Lindstrom, M.F. Sanner, R.K. Belew, D.S. Goodsell, A.J. Olson, *J. Comput. Chem.* **2009**, *16,* 2785-91.

[8] D.A. Case, I.Y. Ben-Shalom, S.R. Brozell, D.S. Cerutti, T.E. Cheatham III, V.W.D. Cruzeiro, T.A. Darden, R.E. Duke, D. Ghoreishi, M.K. Gilson, et al. AMBER 2018, University of California, **2018**.

[9] Woods Group, GLYCAM Web. Complex Carbohydrate Research Center, University of Georgia, Athens, GA, can be found underhttp://www.glycam.com, **2005-2021**.

[10] Protein Preparation Wizard; Epik, Schrödinger, LLC, New York, NY (USA), **2021**; Impact, Schrödinger, LLC, New York, NY (USA), **2021**; Prime, Schrödinger, LLC, New York, NY (USA), **2021**.

[11] D.R. Roe, T.E. Cheatham, 3rd PTRAJ and CPPTRAJ: Software for Processing and Analysis of Molecular Dynamics Trajectory Data. J. Chem. Theory Comput. **2013**, *9,* 3084–3095.

[12] J. Wang, W. Wang, P.A. Kollman, D.A. Case, *J. Mol. Graphics Modell.* **2006**, *25,* 247–260.

[13] W. Humphrey, A. Dalke, K. Schulten, *J. Mol. Graphics* **1996**, *14,* 33–28.
